# Supplementary material for: Involvement of Mitochondria in the Selective Response to Microsecond Pulsed Electric Fields on Healthy and Cancer Stem Cells in the Brain
Source: Int J Mol Sci. 2024 Feb 13;25(4):2233. doi: 10.3390/ijms25042233 (PMC10889160; doi:10.3390/ijms25042233)
Supplement: Supplementary file 1 [file ijms-25-02233-s001.zip › Supplementary Figure S1.pdf]

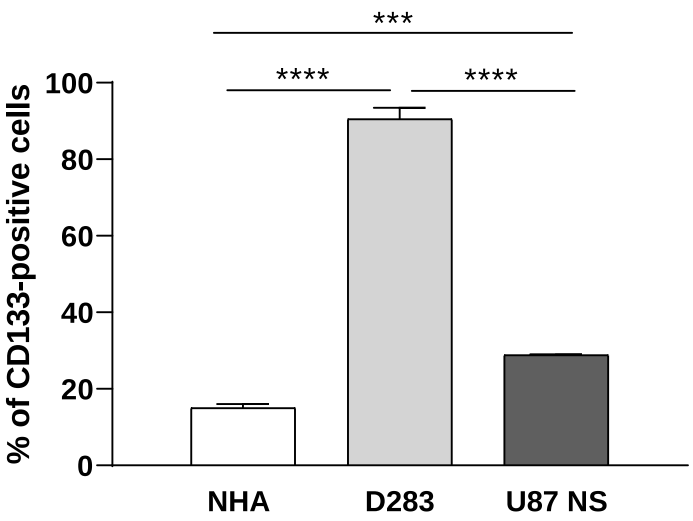

Supplementary Figure S1. Cytofluorimetric quantification of CD133-positive cells in NHA, D283 cells and in U87 NS.
