# Supplementary material for: Involvement of Mitochondria in the Selective Response to Microsecond Pulsed Electric Fields on Healthy and Cancer Stem Cells in the Brain
Source: Int J Mol Sci. 2024 Feb 13;25(4):2233. doi: 10.3390/ijms25042233 (PMC10889160; doi:10.3390/ijms25042233)
Supplement: Supplementary file 1 [file ijms-25-02233-s001.zip › Supplementary Figure S2.pdf]

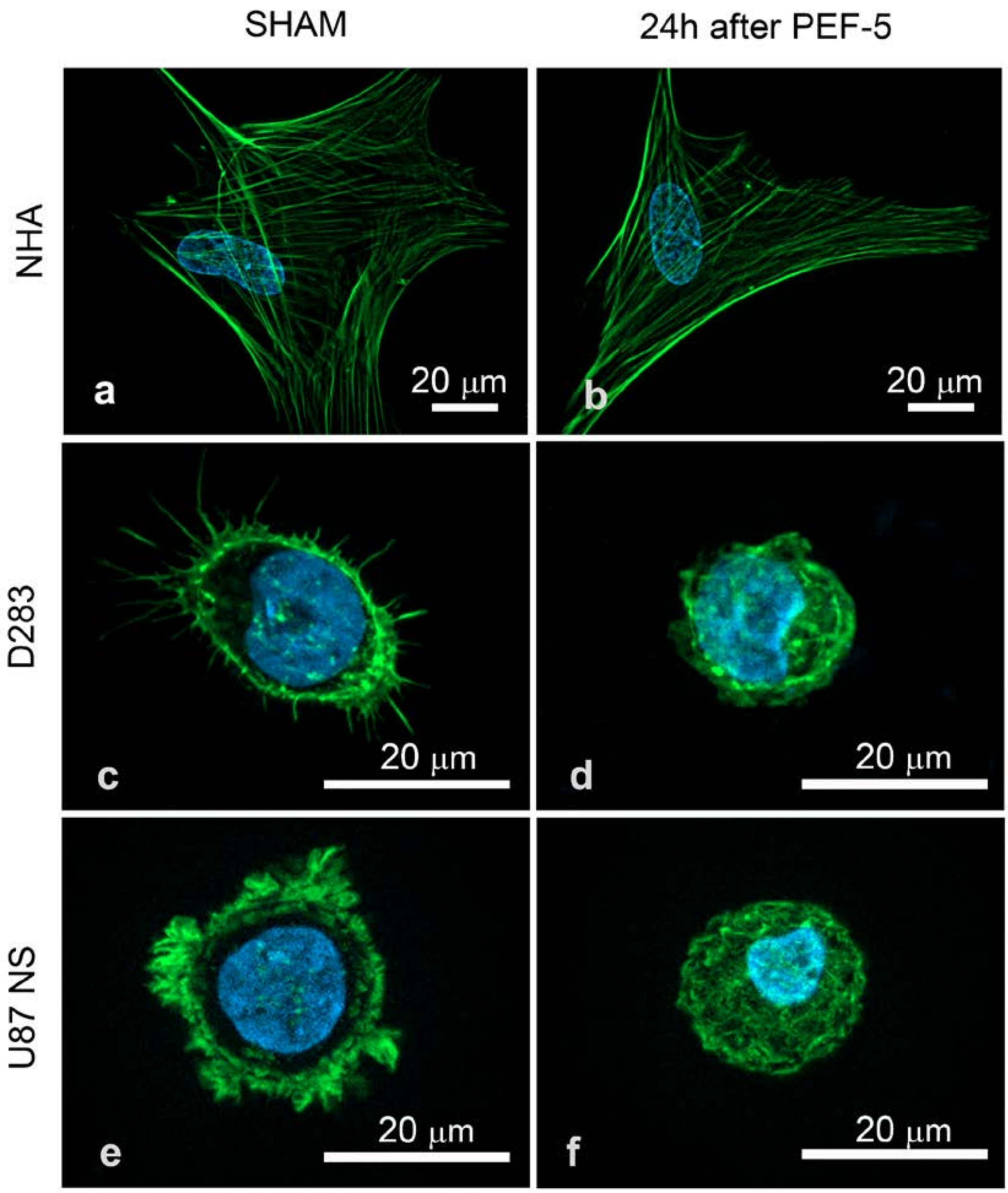

Supplementary Figure S2. Cytoskeleton analysis 24h after PEF-5 exposure. Fluorescent F-actin staining by Alexa Fluor 488 phalloidin highlights cytoskeleton distribution in SHAM- or PEF-5-exposed (a-b) NHA, (c-d) D283 and (e-f) U87 NS respectively.
