# Supplementary material for: Involvement of Mitochondria in the Selective Response to Microsecond Pulsed Electric Fields on Healthy and Cancer Stem Cells in the Brain
Source: Int J Mol Sci. 2024 Feb 13;25(4):2233. doi: 10.3390/ijms25042233 (PMC10889160; doi:10.3390/ijms25042233)
Supplement: Supplementary file 1 [file ijms-25-02233-s001.zip › Supplementary Figure S3.pdf]

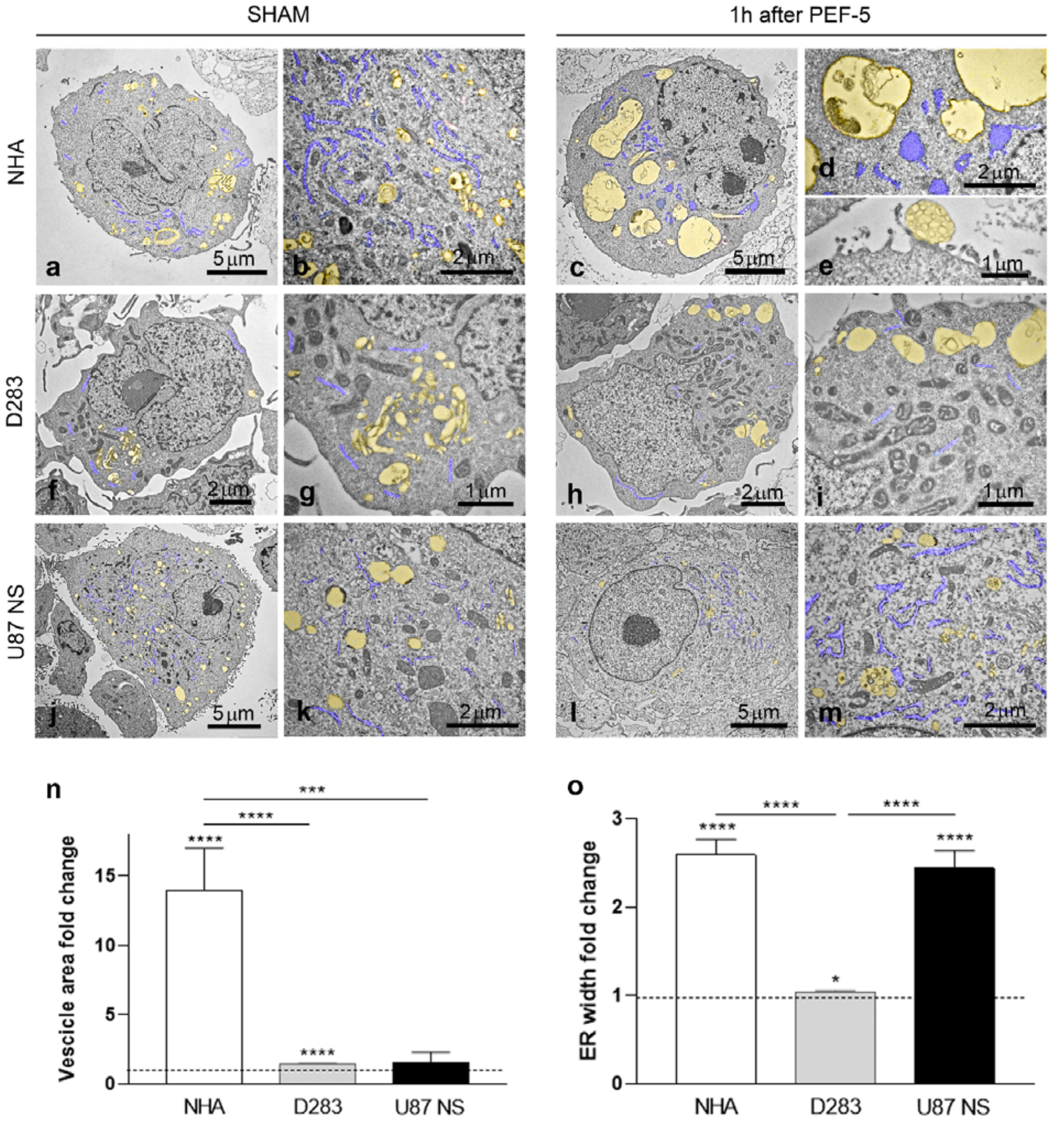

Supplementary Figure S3. Morphological analysis of MVBs formation and ER enlargement. (a-m) TEM images highlights in blue the RE and in yellow the MVBs in (a-b) SHAM-exposed NHA with respect to (c-d) PEF-5-exposed NHA, in (f-g) SHAM-exposed D283 cells with respect to (h-i) PEF-5-exposed D283 cells, in (j-k) SHAM-exposed U87 NS with respect to (l-m) PEF-5-exposed U87 NS. (e) multivesicular bodies are showed on the cell surface of PEF-5-exposed NHA. Graphs show (n) the vesicles area and (o) ER width evaluation expressed as fold increase of PEF-5-exposed cells with respect to relative SHAM (dotted lines).
