# Supplementary material for: Involvement of Mitochondria in the Selective Response to Microsecond Pulsed Electric Fields on Healthy and Cancer Stem Cells in the Brain
Source: Int J Mol Sci. 2024 Feb 13;25(4):2233. doi: 10.3390/ijms25042233 (PMC10889160; doi:10.3390/ijms25042233)
Supplement: Supplementary file 1 [file ijms-25-02233-s001.zip › Supplementary Table S1.pdf]

Supplementary Table S1. List of single-gene fold changes with p value obtained from a whole transcriptome analysis conducted using Clariom S Affymetrix chips in NHA, D283 cells and U87 NS, 24h after PEF-5 exposure.

NHA

Affymetrix_ID	GeneSymbol	logFC (PEF/Sham)	adj.P.Val
TC1200006896.hg.1	GPRC5A; MIR614	1.368285	0.093301
TC2000008318.hg.1	FERMT1	1.085343	0.093301
TC0700011658.hg.1	SEMA3E	0.9285575	0.093301
TC0X00007190.hg.1	PORCN	0.87634	0.093301
TC0100011406.hg.1	CD55	0.73662	0.093301
TC1200009101.hg.1	TMEM233	0.7133175	0.093301
TC0900009569.hg.1	NFIB	-0.6215175	0.093301
TC0100016199.hg.1	OLFML2B	-1.1368425	0.093301

D283

Affymetrix_ID	GeneSymbol	logFC (PEF/Sham)	adj.P.Val
TC0900007576.hg.1	ANXA1	5.096802	0.000808
TC2200007204.hg.1	HMOX1	3.08969	0.008043
TC0X00007919.hg.1	SRPX2	2.479953	0.002284
TC1700008263.hg.1	ABCC3	2.32651	0.000808
TC1200006909.hg.1	EMP1	2.165888	0.009372
TC0500012519.hg.1	SPARC	2.004983	0.016237
TC1600010732.hg.1	NQO1	2.000457	0.001628
TC0300012243.hg.1	SLC12A8	1.979453	0.019885
TC1300009348.hg.1	SPRY2	1.978242	0.000808
TC0500012312.hg.1	SPRY4	1.947323	0.011771
TC1400009214.hg.1	BMP4	1.875035	0.001731
TC1700008228.hg.1	ITGA3	1.79297	0.040728
TC0100013581.hg.1	SPOCD1	1.7608275	0.000808
TC1200007804.hg.1	METTL7B	1.726843	0.009372
TC1000008891.hg.1	DUSP5	1.706438	0.004876
TC0200015958.hg.1	DNER	1.705145	0.008995
TC0300014082.hg.1	ETV5	1.674613	0.002028
Unmapped00000571.hg	GREM1	1.668335	0.017866
TC0400008007.hg.1	GPAT3	1.639177	0.014734
TC0300009855.hg.1	IL1RAP	1.629743	0.002032
TC0900011305.hg.1	TNC	1.599238	0.019857
TC1200011470.hg.1	DUSP6	1.578512	0.000808
TC1100009330.hg.1	UBASH3B	1.54566	0.015832
TC0100009364.hg.1	CSF1	1.542675	0.005559
TC0300009916.hg.1	HES1	1.532572	0.000808
TC1600006652.hg.1	TNFRSF12A	1.519975	0.000808
TC0X00011211.hg.1	G6PD	1.50711	0.000808
TC0300013513.hg.1	P3H2	1.49009	0.005816
TC0700006913.hg.1	GPNMB	1.484563	0.023634
Unmapped00000594.hg	GREM1	1.482605	0.017853
TC1800009290.hg.1	MYO5B	1.460568	0.029311
TC0800010002.hg.1	DUSP4	1.402105	0.009372
TC1100011110.hg.1	CHRM1	1.367577	0.001451
TC0100015925.hg.1	KCNN3	1.3520775	0.051357
TC0800009961.hg.1	CLU; MIR6843	1.32036	0.001682
TC1500007346.hg.1	CGNL1	1.318723	0.067761
TC0200015951.hg.1	PID1	1.312488	0.002284
TC0200012656.hg.1	EFEMP1	1.300445	0.039823
TC2200009234.hg.1	SPECC1L-ADORA2A	1.26284	0.003664
TC0300008467.hg.1	ARHGAP31	1.256165	0.017943
TC1700009731.hg.1	GAS7	1.2507	0.039823
TC0100018355.hg.1	RHOU	1.226305	0.006372
TC1400009529.hg.1	ACTN1; HMGN1P3	1.207155	0.008006
TC0200008900.hg.1	TMEM87B	1.19422	0.001451
TC1000008054.hg.1	PLAU	1.176215	0.008392
TC1600009349.hg.1	GRIN2A	1.174157	0.034346
TC0100016631.hg.1	RGS16	1.17329	0.056365
TC0100015867.hg.1	S100A3	1.152035	0.008043

TC1700010790.hg.1	ETV4	1.150027	0.006068
TC1500006788.hg.1	GREM1	1.139938	0.009488
TC1400009329.hg.1	RTN1	1.124238	0.031761
TC0300010930.hg.1	SLC6A20	1.108898	0.009372
TC0100012921.hg.1	DHRS3; MIR6730	1.108495	0.012849
TC0100016350.hg.1	CCDC181	1.091675	0.003487
TC1900008057.hg.1	ZFP36	1.074825	0.005559
TC0600014265.hg.1	CLIC1	1.07044	0.002032
TC1000007890.hg.1	HKDC1	1.069615	0.004849
TC0500008785.hg.1	EGR1	1.06633	0.034393
TC0100011463.hg.1	SYT14	1.055965	0.015386
TC0700011809.hg.1	TFPI2	1.053597	0.029311
TC2000009458.hg.1	NFATC2	1.046617	0.011967
TC0X00009386.hg.1	SRPX	1.045275	0.072053
TC0500011502.hg.1	ELL2	1.039438	0.022581
TC0400011920.hg.1	SLC7A11	1.038572	0.022541
TC0100013872.hg.1	EDN2	1.032205	0.042782
TC1200011838.hg.1	CMKLR1	1.031098	0.001451
TC1000007895.hg.1	TSPAN15	1.023375	0.003778
TC0100012008.hg.1	KCNK1	1.0168625	0.069123
TC0300007645.hg.1	PTPRG	1.01362	0.08515
TC0200014414.hg.1	CXCR4	1.010835	0.042782
TC0200010216.hg.1	ITGAV	1.00382	0.031042
TC0100007449.hg.1	SH3BGRL3	1.0020925	0.005864
TC0100012006.hg.1	KCNK1	1.001475	0.002731
TC0100010152.hg.1	LMNA	0.99782	0.009372
TC1700009528.hg.1	CXCL16	0.98979	0.00672
TC0600011386.hg.1	IER3	0.9840625	0.002555
TC0100007512.hg.1	GPR3	0.9806875	0.063352
TC1000007891.hg.1	HK1	0.949875	0.042782
TC0200010745.hg.1	IGFBP2	0.9437475	0.047411
TC0600012434.hg.1	ME1	0.9383075	0.010408
TC1100012130.hg.1	MMP10	0.9314475	0.009752
TC0100009572.hg.1	FAM46C	0.930155	0.031891
TC0100018356.hg.1	RHOU	0.928805	0.020367
TC1100012131.hg.1	MMP1	0.9268725	0.048814
TC1800006650.hg.1	RAB31	0.9249975	0.034655
TC1200007992.hg.1	SRGAP1	0.9144775	0.028639
TC0200007803.hg.1	LGALSL	0.9118325	0.016212
TC0300009353.hg.1	PPM1L	0.8798675	0.006916
TC0500012599.hg.1	ADAM19	0.879225	0.002032
TC1200010655.hg.1	LIMA1	0.8762625	0.013435
TC1400009766.hg.1	IRF2BPL	0.8711525	0.020484
TC0900011120.hg.1	KLF4	0.86275	0.018497
TC0600008109.hg.1	VEGFA	0.8573475	0.008043
TC0100018246.hg.1	LRRC8C	0.8533725	0.009083
TC2200007150.hg.1	TIMP3	0.85093	0.002555
TC0600007552.hg.1	DDR1; MIR4640	0.85047	0.005476
TC0300013877.hg.1	EPHB1	0.84744	0.056379
TC1400007755.hg.1	SAMD15	0.84706	0.009083

TC0500012238.hg.1	HBEGF	0.8424975	0.009083
TC0800011683.hg.1	FBXO32	0.83826	0.040454
TC0600007257.hg.1	TRIM38	0.836025	0.039823
TC0100015288.hg.1	KCND3	0.82999	0.029311
TC0100016112.hg.1	TAGLN2	0.8297875	0.022519
TC1800008952.hg.1	DSEL	0.82944	0.029311
TC0X00011404.hg.1	IDS	0.825775	0.029311
TC1600007964.hg.1	MT1B; MT1CP	0.8257625	0.060134
TC1500006925.hg.1	THBS1	0.82378	0.055969
TC2200007439.hg.1	MCHR1	0.8196225	0.026984
TC0800012393.hg.1	ADRA1A	0.815555	0.045988
TC0100016035.hg.1	ETV3	0.813525	0.013906
TC1700007262.hg.1	MAP2K3	0.81221	0.015734
TC1800009245.hg.1	SERPINB8	0.811325	0.026119
TC0100017420.hg.1	DUSP10	0.8106	0.005816
TC1000008489.hg.1	LGI1	0.799655	0.015924
TC1300007491.hg.1	KLF5	0.7985225	0.056409
TC0100011770.hg.1	EPHX1	0.7984325	0.020484
TC1600007962.hg.1	MT1A	0.797865	0.063634
TC1500010350.hg.1	NTRK3	0.792745	0.089615
TC0900010907.hg.1	HSD17B3	0.78805	0.070844
TC2100008496.hg.1	SLC5A3; MRPS6	0.78336	0.009372
TC0700007034.hg.1	CREB5	0.7809075	0.052377
TC0800008243.hg.1	PDP1	0.780705	0.008043
TC1200010866.hg.1	CD63	0.7789725	0.00672
TC0500008568.hg.1	SLC12A2	0.778095	0.054722
TC0800008667.hg.1	NOV	0.7685375	0.057653
TC0900007807.hg.1	DAPK1	0.7676325	0.017168
TC0300010632.hg.1	OSBPL10	0.76559	0.040117
TC0400012820.hg.1	FGF5	0.760355	0.043036
TC1900011774.hg.1	EMP3	0.75618	0.098498
TC1600011399.hg.1	MT1X	0.75496	0.032167
TC0800011075.hg.1	TMEM55A	0.7478275	0.054722
TC0800007114.hg.1	SCARA3	0.743085	0.010829
TC1500010796.hg.1	SYNM	0.741545	0.031042
TC0200008894.hg.1	MERTK	0.7412375	0.030834
TC0500009264.hg.1	ADRA1B	0.74022	0.013638
TC1600007957.hg.1	MT2A	0.7382425	0.026606
TC1400008013.hg.1	SLC24A4	0.7375375	0.095993
TC0300007237.hg.1	LIMD1	0.7365575	0.087317
TC0300006483.hg.1	BHLHE40	0.73412	0.039823
TC0900009949.hg.1	TPM2	0.732285	0.003778
TC0200014361.hg.1	LYPD1	0.73103	0.029311
TC0200015128.hg.1	PDE1A	0.7308225	0.03996
TC1400007566.hg.1	SMOC1	0.72706	0.005559
TC0500009706.hg.1	SQSTM1	0.726985	0.013855
TC1600011440.hg.1	OSGIN1	0.7257175	0.031058
TC0500012826.hg.1	SH3PXD2B	0.7253275	0.015902
TC0100014966.hg.1	GCLM	0.7206725	0.055244
TC0500007201.hg.1	OSMR	0.7149475	0.039823

TC0400010785.hg.1	IGFBP7	0.709805	0.092749
TC0200008230.hg.1	TMSB10	0.7045	0.009752
TC0200009060.hg.1	STEAP3	0.7033825	0.034185
TC1700007135.hg.1	TRIM16L	0.7020675	0.032167
TC1400008486.hg.1	CRIP2	0.7004775	0.015386
TC0900010462.hg.1	RFK	0.6969375	0.005559
TC1200008488.hg.1	PLXNC1	0.6962525	0.019885
TC0100018497.hg.1	SHC1	0.6957625	0.008043
TC1600007967.hg.1	MT1IP	0.68971	0.064187
TC1900006471.hg.1	HCN2	0.6867475	0.048814
TC1200009722.hg.1	TNFRSF1A	0.6802625	0.040396
TC1900007384.hg.1	GDF15	0.6781	0.013982
TC0X00007918.hg.1	TNMD	0.6751175	0.081193
TC1000008315.hg.1	BMPR1A	0.6737975	0.008237
TC0200014620.hg.1	CACNB4	0.67135	0.013435
TC1100012949.hg.1	IFITM1	0.666145	0.015832
TC1100006879.hg.1	PARVA	0.6635025	0.029536
TC0500009544.hg.1	MSX2	0.66324	0.047411
TC1700012039.hg.1	0130370; RP11-10	0.6632025	0.022805
TC1200011474.hg.1	ATP2B1	0.6629675	0.089615
TC1900008432.hg.1	EHD2	0.6607525	0.034718
TC1000006652.hg.1	PFKFB3	0.6560825	0.076954
TC0200007237.hg.1	CRIM1	0.65408	0.083707
TC1600010421.hg.1	MT1G	0.653945	0.058214
Unmapped00000622.hg	GREM1	0.652085	0.020754
TC0200016571.hg.1	OSBPL6	0.650405	0.033907
TC1000007132.hg.1	BAMBI	0.649255	0.070844
TC1700011060.hg.1	FAM117A	0.6445975	0.020754
TC0400012641.hg.1	PDLIM3	0.64399	0.014123
TC1100011052.hg.1	FADS3	0.643235	0.036059
TC1400007201.hg.1	CDKN3	0.640865	0.014734
TC2100007917.hg.1	KRTAP11-1	0.639005	0.083707
TC0500013059.hg.1	CBY3	0.6386425	0.089337
TC0600012542.hg.1	UBE2J1	0.63611	0.013435
TC1200012234.hg.1	CLIP1	0.6341075	0.029311
TC1400009524.hg.1	ZFP36L1	0.6319475	0.020484
TC1700009333.hg.1	NXN	0.6284425	0.033907
TC0600013186.hg.1	CTGF	0.62806	0.093575
TC0400009322.hg.1	GALNT7	0.6280075	0.089615
TC1500010369.hg.1	MFGE8	0.6267775	0.063352
TC1200007859.hg.1	NAB2	0.6260275	0.015832
TC0400012821.hg.1	C4orf22	0.623755	0.047361
TC1900009754.hg.1	DNASE2	0.623495	0.008995
TC1700011074.hg.1	DLX3	0.6225375	0.016237
TC1600007966.hg.1	MT1H	0.6224825	0.063864
TC0700010719.hg.1	TBX20	0.6222575	0.007953
TC2100007803.hg.1	APP	0.6221525	0.014815
TC1900010706.hg.1	SERTAD1	0.6187925	0.048814
TC1900008416.hg.1	INAFM1	0.618225	0.019857
TC0900011938.hg.1	C9orf116	0.61488	0.053736

TC0800011634.hg.1	SNTB1	0.6128175	0.035301
TC0300013684.hg.1	TFRC	0.6120275	0.010786
TC0100009452.hg.1	FAM19A3	0.6113625	0.01441
TC0600012040.hg.1	TRAM2	0.61054	0.079699
TC1900009369.hg.1	PLIN3	0.60819	0.028639
TC0900011379.hg.1	MEGF9	0.6075175	0.095007
TC1700012310.hg.1	MFSD11	0.60698	0.039823
TC1400009151.hg.1	TRIM9	0.60385	0.051499
TC0800009529.hg.1	PPP1R3B	0.5950325	0.023634
TC0600012554.hg.1	BACH2	0.5933475	0.042799
TC0800011334.hg.1	KLF10	0.59026	0.019885
TC0100011920.hg.1	GALNT2	0.588315	0.043968
TC1400007665.hg.1	BBOF1	0.58761	0.021976
TC0300011200.hg.1	TKT	0.5864425	0.021503
Unmapped00000328.hg	DGKD	0.5857275	0.083707
TC1100007884.hg.1	SLC3A2	0.5849475	0.028639
TC1100012686.hg.1	ESAM	0.5826525	0.040117
TC1900008507.hg.1	FTL	0.58121	0.040148
TC1200010865.hg.1	ITGA7	0.58007	0.019885
TC0600008146.hg.1	RUNX2	0.578985	0.01049
TC0600010853.hg.1	GFOD1	0.578025	0.038333
TC0500010635.hg.1	HMGCS1	0.5725125	0.09986
TC1200012641.hg.1	RDH5	0.5724875	0.042782
TC1200008675.hg.1	TXNRD1	0.572415	0.035368
TC0300014090.hg.1	ATP13A3	0.5713275	0.021976
TC1800008287.hg.1	ANKRD29	0.56696	0.061048
TC1700006627.hg.1	SPNS2	0.56536	0.087532
TC0300012238.hg.1	HEG1	0.56459	0.031307
TC1600010603.hg.1	RRAD	0.5643	0.028639
TC0300012233.hg.1	ITGB5	0.5641525	0.021679
TC0900012157.hg.1	AKAP2	0.56053	0.039156
TC2100007037.hg.1	CBR3	0.558625	0.036649
TC1400010616.hg.1	SLC38A6	0.5498525	0.025274
TC0100008017.hg.1	RIMKLA	0.5481725	0.080167
TC0300010975.hg.1	KIF9	0.5474125	0.093525
TC0500007258.hg.1	GHR	0.54726	0.063597
TC1100009533.hg.1	ADAMTS15	0.546845	0.099937
TC1700011774.hg.1	TRIM47	0.5429975	0.060052
TC1000011606.hg.1	BLOC1S2	0.5411175	0.015386
TC0700008306.hg.1	FZD1	0.54058	0.07088
TC0100009908.hg.1	ECM1	0.5397825	0.022541
TC1000010172.hg.1	SVIL	0.5386525	0.076511
TC0100010887.hg.1	C1orf21	0.5366675	0.079459
TC2100007140.hg.1	ETS2	0.535835	0.04219
TC0100013713.hg.1	STK40	0.5351975	0.040148
TC1900007127.hg.1	IER2	0.5334775	0.029311
TC0100017575.hg.1	WNT9A	0.533355	0.05439
TC0300009324.hg.1	J; SCHIP1; IQCJ-SCH	0.5328575	0.029311
TC0300011525.hg.1	RYBP	0.532135	0.093575
TC1000010006.hg.1	DNAJC1	0.5261625	0.033907

TC0300007257.hg.1	CCR5	0.5257175	0.037257
TC1500008757.hg.1	ATP10A	0.523945	0.083402
TC1100013097.hg.1	REXO2	0.52005	0.090778
TC1900011663.hg.1	CCDC159	0.5194475	0.096851
TC0X00010877.hg.1	FAM122B	0.519055	0.020484
TC2200009236.hg.1	GGT1	0.51659	0.039397
TC0100006773.hg.1	SPSB1	0.51646	0.015622
TC1400007710.hg.1	JDP2	0.514905	0.019857
TC1700007791.hg.1	RARA	0.5148075	0.044279
TC1700008216.hg.1	KAT7	0.50975	0.05125
TC0300008550.hg.1	CSTA	0.50632	0.083707
TC1000008056.hg.1	VCL	0.50526	0.036649
Unmapped00000639.hg	SERTAD4	0.5031725	0.087852
TC0300013360.hg.1	ABCC5	0.5028875	0.052322
TC1100006494.hg.1	CD151	0.5020975	0.032167
TC1800007276.hg.1	CTIF; MIR4743	0.501635	0.045988
TC0400010688.hg.1	USP46	0.50094	0.049675
TC1600007965.hg.1	MT1F	0.5008575	0.063352
TC1800008821.hg.1	RAX	0.49594	0.042782
TC0500013064.hg.1	MGAT4B	0.4945525	0.063352
TC1700012361.hg.1	CDRT1	0.4921375	0.086088
TC0100007370.hg.1	RCAN3	0.4913225	0.056715
TC0200013135.hg.1	EVA1A	0.4895425	0.081553
TC0800007185.hg.1	RBPMS	0.4884475	0.073421
TC0200010858.hg.1	STK11IP	0.48659	0.054881
TC0200016597.hg.1	SPEG	0.4856775	0.091951
TC1900006499.hg.1	ARID3A	0.485395	0.013857
TC0X00011382.hg.1	ACSL4	0.4839225	0.089988
TC1900011867.hg.1	TMEM205	0.4804425	0.065308
TC1700007677.hg.1	DUSP14	0.47662	0.067922
TC1100008469.hg.1	SERPINH1	0.4759825	0.08467
TC0300014093.hg.1	PCYT1A	0.4748325	0.026099
TC0800010034.hg.1	GSR	0.474415	0.059872
TC0X00007507.hg.1	EFNB1	0.4742125	0.055098
TC1400010195.hg.1	WARS	0.473935	0.061412
TC0100013028.hg.1	EPHA2	0.470065	0.081684
TC1200008683.hg.1	CHST11	0.4699375	0.032144
TC1200010493.hg.1	DBX2	0.46939	0.034185
TC0900008109.hg.1	HABP4	0.4690475	0.029311
TC0400006583.hg.1	GRK4	0.466845	0.039397
TC1900011958.hg.1	ERF	0.46413	0.040117
TC1700006660.hg.1	KIF1C	0.4637875	0.037923
TC0200011054.hg.1	ARMC9	0.4613125	0.08286
TC0900012162.hg.1	ORM1	0.4589625	0.059063
TC1100007577.hg.1	TRIM48	0.4571225	0.069087
TC1000012423.hg.1	PFKP	0.453035	0.026119
TC0800007367.hg.1	ADAM9	0.45202	0.083707
TC1300009538.hg.1	OXGR1	0.44903	0.089615
TC0100010104.hg.1	ZBTB7B	0.44829	0.043968
TC2200009233.hg.1	ADORA2A	0.4470025	0.052426

TC1000006802.hg.1	CAMK1D	0.4455475	0.083408
TC1100009335.hg.1	C11orf63	0.4452775	0.095599
TC1000007990.hg.1	DDIT4	0.44519	0.084384
TC0600011376.hg.1	PPP1R18	0.444865	0.072647
TC0X00008198.hg.1	PLS3	0.4442525	0.041902
TC0700008563.hg.1	EPO	0.44271	0.049675
TC0X00010356.hg.1	BTK	0.4420925	0.072053
TC0400012621.hg.1	ACSL1	0.4416425	0.089988
TC1600007960.hg.1	MT1M	0.44157	0.085213
TC1100011295.hg.1	CCDC87	0.4415375	0.032939
TC1700012370.hg.1	TBC1D28	0.440995	0.068578
TC1100011652.hg.1	GDPD5	0.438865	0.063864
TC1100009521.hg.1	APLP2	0.4383925	0.062066
TC0200006779.hg.1	FAM84A	0.437425	0.050232
TC0800008783.hg.1	SQLE	0.4365325	0.048814
TC2000006559.hg.1	CDC25B	0.4338375	0.069087
TC0900010971.hg.1	TBC1D2	0.43239	0.087317
TC0300007220.hg.1	ZNF502	0.4322275	0.096313
TC0800012290.hg.1	UBXN8	0.4317825	0.097452
TC0200013298.hg.1	ST3GAL5	0.431575	0.099173
TC0X00008833.hg.1	FAM50A	0.43152	0.054722
TC2100007508.hg.1	KCNE1	0.430735	0.083707
Unmapped00000145.hg	SAG	0.4290025	0.091951
TC0100018463.hg.1	RHOC	0.42836	0.038773
TC0700006629.hg.1	FSCN1	0.4280175	0.091951
TC2200006501.hg.1	IL17RA	0.427245	0.084479
TC1200008924.hg.1	DTX1	0.4251675	0.069123
TC2200008966.hg.1	KIAA0930	0.42494	0.084891
TC2100008417.hg.1	COL18A1-AS1	0.424675	0.061141
TC1200012597.hg.1	MGST1	0.4229825	0.042564
TC1500006896.hg.1	SPRED1	0.42189	0.055828
TC1400010033.hg.1	ASB2	0.4190025	0.067659
TC1100013192.hg.1	CLCF1	0.411835	0.07981
TC1800006506.hg.1	MYL12A	0.4118075	0.075624
TC0500012938.hg.1	CLTB	0.41161	0.037257
TC1900011331.hg.1	ZNF665	0.40821	0.073421
TC2200007246.hg.1	MYH9	0.40808	0.043948
TC0900011404.hg.1	STOM	0.4075	0.065308
TC0200011062.hg.1	B3GNT7	0.40729	0.089722
TC1100011451.hg.1	FGF4	0.406285	0.039397
TC0600011065.hg.1	KIAA0319	0.4056	0.09271
TC1200011310.hg.1	PHLDA1	0.4050425	0.058214
TC0200011130.hg.1	DGKD	0.404785	0.062916
TC0600011809.hg.1	CCND3	0.40398	0.08302
TC0600007477.hg.1	MOG	0.40262	0.081193
TC0500009346.hg.1	WWC1	0.40221	0.088133
TC1200010158.hg.1	KRAS	0.4004875	0.09271
TC0100011721.hg.1	CAPN2	0.4002425	0.056379
TC1000012582.hg.1	RRP12	0.399745	0.099979
TC0100010577.hg.1	MROH9	0.39919	0.054722

;Unmapped00000098.hg	DGKD	0.3974525	0.061889
TC1200010653.hg.1	CERS5	0.39564	0.049941
TC0100017212.hg.1	SLC30A1	0.392975	0.070844
TC0400008616.hg.1	BBS12	0.38977	0.090101
TC1700009385.hg.1	SLC43A2	0.3897275	0.063352
TC0600014215.hg.1	GMDS	0.385335	0.095007
TC1900008184.hg.1	CIC	0.385205	0.067922
TC0700006915.hg.1	MALSU1	0.3839975	0.087852
TC0800007245.hg.1	MAK16	0.3837225	0.045988
TC0300006687.hg.1	CCDC174	0.3833175	0.054722
TC0300008964.hg.1	MRPS22	0.3758225	0.081553
TC0100016296.hg.1	GPA33	0.3753975	0.047411
TC1200012153.hg.1	PXN	0.37468	0.095007
TC0700013341.hg.1	CHN2	0.370945	0.089615
TC0600012871.hg.1	TRAF3IP2	0.36989	0.083707
TC1600008141.hg.1	CBFB	0.368255	0.05933
TC1600006641.hg.1	FLYWCH2	0.3676425	0.063528
TC0200016570.hg.1	HOXD3	0.36505	0.063352
TC1900006588.hg.1	GADD45B	0.365	0.079699
TC1200010145.hg.1	BCAT1	0.36338	0.072053
TC1700008945.hg.1	SPHK1	0.3625075	0.079699
TC0900007064.hg.1	DNAJB5	0.362205	0.09375
TC0200008617.hg.1	PDCL3	0.361595	0.070844
TC1100010051.hg.1	TMEM9B	0.3612575	0.073139
TC2200008467.hg.1	PLA2G3	0.3595275	0.069087
TC0900008869.hg.1	ODF2	0.3594225	0.057268
TC0900011177.hg.1	TXN	0.3542575	0.061048
TC0800010275.hg.1	PLAT	0.3542225	0.08515
TC1100011058.hg.1	FTH1	0.3540125	0.057585
TC0300013934.hg.1	EMC3	0.35272	0.087258
;Unmapped00000001.hg	ZKSCAN7	0.351625	0.071512
TC1300008108.hg.1	F7	0.350515	0.077921
TC1800006757.hg.1	LDLRAD4	0.350515	0.089988
TC0300013874.hg.1	SRPRB	0.349685	0.079699
TC0X00010880.hg.1	MOSPD1	0.34955	0.081684
TC0500010835.hg.1	PLK2	0.3455325	0.065979
TC0100015196.hg.1	PSMA5	0.3431525	0.065529
;Unmapped00000819.hg	PCYT1A	0.3410525	0.083707
TC1000011673.hg.1	PITX3	0.3404625	0.07981
TC0700009696.hg.1	CHPF2; MIR671	0.3385775	0.095007
TC1200008107.hg.1	RAP1B	0.33849	0.068526
TC0500008849.hg.1	CYSTM1	0.3313375	0.06381
TC0800006873.hg.1	PDGFRL	0.3303525	0.082814
TC0400008086.hg.1	IBSP	0.32945	0.088133
TC0200007477.hg.1	SOCS5	0.3288325	0.092
TC1900011114.hg.1	LHB	0.3214525	0.089722
TC0900010661.hg.1	CDK20	0.3184675	0.099735
TC1700011233.hg.1	MRPS23	0.3176525	0.073421
;Unmapped00000525.hg	BCL2L14	0.3168325	0.090037
TC0200016580.hg.1	C2orf88	0.3149975	0.089615

TC0X00009695.hg.1	LINC01496	0.31016	0.096085
TC1200012642.hg.1	S1-RDH5; RP11-64	0.3059575	0.095007
TC0800006975.hg.1	BMP1	0.3019175	0.091951
TC0100007940.hg.1	CAP1	0.2908725	0.089615
TC1000011026.hg.1	USP54	-0.293345	0.092
TC1200008800.hg.1	UBE3B	-0.29789	0.095007
TC2000009014.hg.1	NDRG3	-0.30312	0.089615
TC0900008286.hg.1	SMC2	-0.3077025	0.095007
TC1500010072.hg.1	SIN3A	-0.3181075	0.092
TC0700012959.hg.1	EZH2	-0.320915	0.064187
TC2000007819.hg.1	CSTF1	-0.3265525	0.088133
TC2100007474.hg.1	PRMT2	-0.331705	0.081662
TC0900012225.hg.1	RNF38	-0.33424	0.081553
TC0X00007205.hg.1	HDAC6	-0.3368325	0.074228
TC0100017451.hg.1	TP53BP2	-0.337715	0.071772
TC0600011225.hg.1	HIST1H2AJ	-0.3383125	0.096517
TC1900007743.hg.1	ZNF507	-0.3389725	0.086099
TC0100018390.hg.1	SLC35E2	-0.3394225	0.086605
TC2000007218.hg.1	CEP250	-0.339945	0.08302
TC0600007268.hg.1	HIST1H4C	-0.341805	0.088857
TC1600007113.hg.1	C16orf62	-0.3485075	0.070844
TC0600011465.hg.1	EHMT2	-0.34879	0.077921
TC0500012162.hg.1	BRD8	-0.353885	0.065308
TC0300011801.hg.1	MINA	-0.35862	0.095007
TC0300011706.hg.1	VGLL3	-0.35959	0.067139
TC1000007925.hg.1	EIF4EBP2	-0.3601875	0.096313
TC0400012945.hg.1	HSD17B11	-0.3606375	0.088133
TC1200010119.hg.1	C2CD5	-0.3617425	0.091951
TC1900011968.hg.1	XRCC1	-0.36363	0.068526
TC1300008426.hg.1	USP12	-0.3637175	0.08286
TC0400006578.hg.1	SH3BP2	-0.36389	0.064187
TC0200015080.hg.1	PRKRA	-0.3644825	0.098507
TC1500010740.hg.1	DTWD1	-0.3681825	0.065308
TC1900008904.hg.1	KMT5C	-0.3696175	0.081662
TC0200010116.hg.1	DFNB59	-0.371265	0.092589
TC0200015431.hg.1	TMEM237	-0.37163	0.073421
TC0600011184.hg.1	HIST1H2BJ	-0.372195	0.087276
TC0X00007889.hg.1	DIAPH2	-0.3724075	0.070844
TC0100016969.hg.1	KLHL12	-0.37273	0.058863
TC0200013531.hg.1	KANSL3	-0.379075	0.056379
TC0200014192.hg.1	SAP130	-0.379785	0.091951
TC1000006917.hg.1	CACNB2	-0.38186	0.098033
TC0900007472.hg.1	ANKRD20A1	-0.385475	0.069123
TC1000011040.hg.1	CAMK2G	-0.3859425	0.06786
TC0600007287.hg.1	HIST1H3E	-0.3862	0.069108
TC0900007115.hg.1	GLIPR2	-0.38626	0.084239
TC0100018388.hg.1	SLC35E2B	-0.3863075	0.063352
TC0400012432.hg.1	HMGB2	-0.38709	0.056379
TC2200007574.hg.1	PARVB	-0.3871525	0.095007
TC1400009912.hg.1	GALC	-0.387905	0.076471

TC1900009198.hg.1	BTBD2	-0.38983	0.039397
TC0800011679.hg.1	ATAD2	-0.39001	0.07611
TC1500006999.hg.1	NUSAP1	-0.3908175	0.053804
TC1400009829.hg.1	CEP128	-0.391325	0.092303
TC0100009876.hg.1	T2H2AA3; HIST2H2	-0.3939625	0.088133
TC0600007868.hg.1	RNF8	-0.39607	0.076471
TC0600011870.hg.1	DNPH1	-0.3961925	0.090736
TC0900009581.hg.1	FREM1	-0.3989875	0.087852
TC1700007469.hg.1	ATAD5	-0.3991525	0.046604
TC1000010917.hg.1	LRRC20	-0.399285	0.088857
TC0100012490.hg.1	C1orf233	-0.3997425	0.09616
TC1900011055.hg.1	BSPH1	-0.40126	0.090593
TC1600011371.hg.1	SLX1A; SLX1B	-0.40195	0.080167
TC2000009780.hg.1	TCFL5	-0.4021625	0.086719
TC1100010856.hg.1	OR5M9	-0.402835	0.06786
TC0100013608.hg.1	MARCKSL1	-0.40343	0.07981
TC1700008331.hg.1	HLF	-0.4039625	0.080167
TC0100008126.hg.1	AKR1A1	-0.4039875	0.096408
TC0400010260.hg.1	SEPSECS	-0.404345	0.048814
TC0600008780.hg.1	CASP8AP2	-0.4043975	0.072372
TC2000007512.hg.1	PCIF1	-0.40518	0.039397
TC0600011233.hg.1	HIST1H3I	-0.40622	0.087532
TC0600007869.hg.1	CMTR1	-0.4066525	0.053882
TC1900012000.hg.1	TEAD2	-0.40679	0.049941
TC1500010731.hg.1	TRIM69	-0.4072825	0.070844
TC0100007716.hg.1	ZNF362	-0.4077525	0.087852
TC2200008610.hg.1	FOXRED2	-0.4078125	0.077921
TC0200007929.hg.1	GMCL1	-0.4079225	0.092
TC0700008755.hg.1	DUS4L	-0.4093425	0.049571
TC0400012769.hg.1	BST1	-0.4096775	0.09271
TC1200010917.hg.1	TIMELESS	-0.411655	0.061412
TC0500007077.hg.1	NPR3	-0.4129075	0.068526
TC1300007248.hg.1	CKAP2	-0.4129575	0.081193
TC1700010618.hg.1	TOP2A	-0.4152875	0.052426
TC0200013535.hg.1	LMAN2L	-0.4200775	0.098507
TC0700011979.hg.1	GPC2	-0.4207025	0.089615
TC1200012573.hg.1	RHNO1	-0.42234	0.043948
TC1700011761.hg.1	RECQL5	-0.42318	0.053736
TC0600007700.hg.1	KIFC1	-0.4271225	0.042782
TC0100011581.hg.1	CENPF	-0.43036	0.057585
TC2000008815.hg.1	BCL2L1	-0.4307275	0.061499
TC0X00010804.hg.1	ZNF280C	-0.4308225	0.081307
TC1400010624.hg.1	PTGR2	-0.4315475	0.082468
TC0300011173.hg.1	NT5DC2	-0.43289	0.082751
TC0600008863.hg.1	MANEA	-0.4331075	0.095007
Unmapped00000242.hg	KIF15	-0.43438	0.053604
TC0800007065.hg.1	CDCA2	-0.43524	0.084578
TC1600008577.hg.1	BCO1	-0.435985	0.090698
TC1100008847.hg.1	CEP57	-0.436105	0.032144
TC0300012718.hg.1	PLOD2	-0.438605	0.086605

TC1500008154.hg.1	ZSCAN2	-0.4398525	0.089615
TC0400007868.hg.1	PARM1	-0.4399375	0.09887
TC0400006994.hg.1	NCAPG	-0.442875	0.075663
TC1200009649.hg.1	CRACR2A	-0.44553	0.042782
TC0900010769.hg.1	NFIL3	-0.44666	0.070681
TC0300013989.hg.1	ABHD14B	-0.4479475	0.06786
TC1300007731.hg.1	GPR180	-0.4483325	0.077921
TC0100016831.hg.1	ASPM	-0.4489325	0.032939
TC1600011007.hg.1	MPHOSPH6	-0.4490475	0.065308
TC0100008260.hg.1	CDKN2C	-0.450245	0.094938
TC1700006592.hg.1	GSG2	-0.4515325	0.070844
TC0X00009890.hg.1	ZC4H2	-0.45266	0.070844
TC0X00006671.hg.1	MOSPD2	-0.45433	0.087276
TC0100013854.hg.1	SCMH1	-0.4570725	0.060361
TC0600007285.hg.1	HIST1H2AE	-0.4589225	0.055325
TC1700009254.hg.1	HEXDC	-0.4608625	0.034155
TC0100015701.hg.1	IIST2H3A; HIST2H3	-0.46108	0.099389
TC0600012631.hg.1	MMS22L	-0.4612375	0.058367
TC0200016561.hg.1	KLHL23	-0.4613425	0.04773
TC1500010755.hg.1	ANKDD1A	-0.462325	0.053804
TC1000008731.hg.1	RPARP-AS1	-0.462325	0.093965
TC0300012140.hg.1	GPR156	-0.462585	0.09986
TC0700012163.hg.1	EFCAB10	-0.462645	0.058214
TC0900009592.hg.1	PSIP1	-0.46296	0.061889
TC0200016563.hg.1	KLHL23	-0.46451	0.044574
TC0200016753.hg.1	CCDC141	-0.46498	0.061141
TC0900008497.hg.1	ZNF618	-0.465475	0.038026
TC2100008467.hg.1	C21orf58	-0.4682975	0.020754
TC1800009268.hg.1	DSC2	-0.46963	0.09271
TC0400007582.hg.1	KIAA1211	-0.470155	0.070844
TC0300011731.hg.1	CGGBP1	-0.471005	0.028639
TC1000010847.hg.1	DNA2	-0.4718975	0.067824
TC0X00006483.hg.1	GYG2	-0.4725775	0.046555
TC0900007953.hg.1	CENPP	-0.4730375	0.07981
TC0900009513.hg.1	TMEM261	-0.47555	0.049941
TC2100007355.hg.1	PFKL	-0.47586	0.055555
TC0900009673.hg.1	MLLT3	-0.475875	0.083707
TC0700011318.hg.1	ERV3-1; ZNF117	-0.4767475	0.044208
TC1600006904.hg.1	RMI2	-0.4768525	0.067139
TC0100016364.hg.1	SCYL3	-0.4769075	0.048589
TC1400008780.hg.1	NOVA1	-0.4770325	0.04756
TC0100011472.hg.1	HHAT	-0.47744	0.048814
TC0900010969.hg.1	CORO2A	-0.4775675	0.081553
TC1500009639.hg.1	ICE2	-0.4796675	0.075663
TC0100011871.hg.1	HIST3H2BB	-0.47976	0.039397
TC0300013103.hg.1	RPL22L1	-0.4798075	0.063634
TC1200012143.hg.1	CIT; MIR1178	-0.4806275	0.086605
TC0300009126.hg.1	GYG1	-0.4807375	0.094546
TC0900012180.hg.1	PHYHD1	-0.4811475	0.083707
TC0500010085.hg.1	CMBL	-0.4822325	0.075569

TC0100018302.hg.1	EFNA4	-0.4823625	0.047126
TC0100018200.hg.1	SEPN1	-0.4831975	0.022581
TC1000007056.hg.1	THNSL1	-0.4840975	0.075569
TC1700010802.hg.1	MPP2	-0.4842025	0.091803
TC0200016684.hg.1	TIA1	-0.4844625	0.056379
TC0600011255.hg.1	ZSCAN23	-0.4846475	0.088127
TC0200015631.hg.1	IKZF2	-0.4853725	0.069087
TC0100009877.hg.1	HIST2H3A	-0.4853775	0.078878
TC1500007392.hg.1	CCNB2	-0.4854475	0.092
TC0X00008055.hg.1	TBC1D8B	-0.48623	0.090736
TC0400011524.hg.1	TBCK	-0.48681	0.047411
TC1700007097.hg.1	GID4	-0.4890375	0.035368
TC0400011477.hg.1	CENPE	-0.489285	0.061048
TC0300012654.hg.1	TFDP2	-0.489395	0.095993
TC1200007499.hg.1	TMEM106C	-0.4903475	0.049182
TC0300007223.hg.1	KIF15	-0.4903875	0.079409
TC1800008399.hg.1	B4GALT6	-0.490875	0.089615
TC1000008471.hg.1	KIF11	-0.4909225	0.044134
TC0600009459.hg.1	ENPP1	-0.49169	0.028639
TC0900012120.hg.1	IL11RA	-0.49301	0.028639
TC0200015869.hg.1	AP1S3	-0.4937475	0.070671
TC1400008688.hg.1	C14orf93	-0.495935	0.078946
TC0600011136.hg.1	HIST1H2BG	-0.4962775	0.059742
Unmapped00000414.hg	TMEM42	-0.4976	0.076471
TC1700009694.hg.1	MYH10	-0.4978075	0.070369
TC0500011924.hg.1	ALDH7A1	-0.498205	0.028639
TC1500007659.hg.1	PIAS1	-0.49851	0.019885
TC0400008331.hg.1	NPNT	-0.4992775	0.067824
TC1900008369.hg.1	HIF3A	-0.4995575	0.083408
TC0100010553.hg.1	C1orf112	-0.50303	0.087338
TC0600011235.hg.1	ST1H2AM; HIST1H	-0.503475	0.087852
TC1100008983.hg.1	ACAT1	-0.50845	0.048589
TC1200006935.hg.1	ATF7IP	-0.5096375	0.09271
TC0X00007936.hg.1	CENPI	-0.509905	0.056664
TC0100015342.hg.1	SYT6	-0.5107325	0.058205
TC0200009687.hg.1	ARL6IP6	-0.51075	0.089615
TC2000008200.hg.1	PCED1A	-0.5120475	0.061499
TC0600010235.hg.1	MLLT4	-0.5131225	0.089722
TC0800006869.hg.1	SLC7A2	-0.513165	0.043948
TC0400009223.hg.1	CPE	-0.513805	0.077005
TC0700008617.hg.1	SH2B2	-0.5142225	0.026984
TC0200010658.hg.1	CPS1	-0.51697	0.095007
TC1000010070.hg.1	PRTFDC1	-0.5171025	0.08062
TC0100009788.hg.1	BCL9	-0.517355	0.027265
TC0600012817.hg.1	AK9	-0.52058	0.0939
TC0600007374.hg.1	HIST1H2AI	-0.52143	0.076471
TC0500013392.hg.1	CCNJL	-0.5220125	0.034346
TC0600011232.hg.1	HIST1H1B	-0.5221	0.061465
TC1200012706.hg.1	ACAD10	-0.5227175	0.047411
TC0100018417.hg.1	ALDH4A1	-0.5229875	0.045766

TC0200016494.hg.1	CNNM4	-0.5242125	0.022541
TC0700010437.hg.1	RAPGEF5	-0.524235	0.03393
TC0100010301.hg.1	VANGL2	-0.5249025	0.081256
TC0500011804.hg.1	DTWD2	-0.5251725	0.028639
TC0600008462.hg.1	COL19A1	-0.52734	0.087532
TC1400008919.hg.1	CFL2	-0.528295	0.09986
TC0900011769.hg.1	FAM78A	-0.5285825	0.065308
TC0200011551.hg.1	ADI1	-0.529505	0.019885
TC1500007612.hg.1	DIS3L	-0.529675	0.054201
TC0600012652.hg.1	FAXC	-0.5298825	0.052483
TC0600014083.hg.1	HIST1H2AG	-0.5323125	0.026119
TC1200009621.hg.1	FOXM1	-0.53304	0.048371
TC0600007274.hg.1	HIST1H2BD	-0.5349775	0.053804
TC0900009231.hg.1	FAM69B	-0.5353675	0.029311
TC2200009336.hg.1	NIPSNAP1	-0.5355725	0.020416
TC0X00010387.hg.1	TMSB15A	-0.53867	0.055814
TC2000008123.hg.1	NRSN2-AS1	-0.5403275	0.035368
TC0X00009057.hg.1	MID1	-0.54404	0.023634
TC1000009919.hg.1	TRDMT1	-0.5451725	0.088133
TC0100007562.hg.1	TRNAU1AP	-0.545625	0.092
TC1900008113.hg.1	LTBP4	-0.546195	0.073126
TC1900006991.hg.1	SLC44A2	-0.5476425	0.023084
TC1200010050.hg.1	LMO3	-0.5487175	0.019885
TC1200012666.hg.1	TMEM19	-0.549385	0.043968
TC0100018285.hg.1	NBPF19	-0.5495725	0.037257
TC0400012458.hg.1	HPGD	-0.5505225	0.037257
TC0600008630.hg.1	TTK	-0.55092	0.081553
TC0500011705.hg.1	NREP	-0.55134	0.021514
TC0100013369.hg.1	STMN1; MIR3917	-0.5514425	0.015902
TC0600007789.hg.1	ZNF76	-0.552015	0.024183
TC0600013602.hg.1	FBXO5	-0.552495	0.043948
TC0600011124.hg.1	HIST1H3B	-0.552555	0.083707
TC0800011158.hg.1	C8orf37	-0.553665	0.087276
TC2200007069.hg.1	OSBP2	-0.5548925	0.04434
TC2100008095.hg.1	PIGP	-0.5579425	0.092
TC1100008819.hg.1	KDM4D	-0.55817	0.038026
TC0X00009867.hg.1	SPIN4	-0.5592275	0.040063
TC0400012251.hg.1	C4orf46	-0.559305	0.076438
TC1200011330.hg.1	E2F7	-0.5614775	0.042782
TC0900011163.hg.1	PTPN3	-0.56193	0.047518
TC1200009676.hg.1	AKAP3	-0.56201	0.068578
TC1700008769.hg.1	KCNJ2	-0.564705	0.049814
TC0X00010837.hg.1	MBNL3	-0.56491	0.039397
TC0600013458.hg.1	EPM2A	-0.566615	0.047361
TC1700009679.hg.1	AURKB	-0.5676475	0.088133
TC1100011742.hg.1	KCTD21	-0.569855	0.063209
TC0600010802.hg.1	ELOVL2	-0.5698775	0.024183
TC1200011327.hg.1	CSRP2	-0.57013	0.043948
TC0500008582.hg.1	ISOC1	-0.5707275	0.032531
TC0500007143.hg.1	SKP2	-0.573355	0.081193

TC1800008070.hg.1	PIEZO2	-0.5748775	0.091951
TC1200007891.hg.1	DTX3	-0.5767925	0.016877
TC1800007411.hg.1	NEDD4L	-0.5780075	0.032144
TC0100008607.hg.1	RAVER2	-0.578775	0.026407
TC0100009870.hg.1	IIST2H4B; HIST2H4	-0.5821925	0.070844
TC1900010360.hg.1	ANKRD27	-0.58267	0.047411
TC0300011314.hg.1	C3orf67	-0.5849625	0.063352
TC1100006773.hg.1	TUB	-0.586995	0.054722
TC1200007630.hg.1	LETMD1	-0.58719	0.043948
TC1400010714.hg.1	HAUS4; MIR4707	-0.5903825	0.04756
TC1100010026.hg.1	LMO1	-0.593495	0.047361
TC0500008437.hg.1	TNFAIP8	-0.5948025	0.04434
TC0600011135.hg.1	ST1H3D; HIST1H2/	-0.6002	0.063352
TC0700013338.hg.1	GLCCI1	-0.6007975	0.08302
TC0100015707.hg.1	IIST2H4A; HIST2H4	-0.60244	0.061048
TC1700011181.hg.1	MMD	-0.604425	0.027997
TC1000006862.hg.1	OLAH	-0.6044475	0.04756
TC0900012155.hg.1	MSANTD3-TMEFF1	-0.6060125	0.028639
TC2200007620.hg.1	FBLN1	-0.6075175	0.033907
TC1600011449.hg.1	SPIRE2	-0.6078375	0.020295
TC0200010907.hg.1	ACSL3	-0.6078375	0.048814
TC1900007113.hg.1	DAND5	-0.61008	0.063352
TC0600011228.hg.1	HIST1H2AK	-0.610305	0.079699
TC0900012154.hg.1	TMEFF1	-0.6116975	0.021503
TC0900007740.hg.1	RMI1	-0.61218	0.028796
TC0400008348.hg.1	HADH	-0.61229	0.022541
TC1100010792.hg.1	FOLH1	-0.6134675	0.080883
TC1200008597.hg.1	GAS2L3	-0.6162675	0.055828
TC1700006611.hg.1	CYB5D2	-0.617635	0.028639
TC0100018440.hg.1	STIL	-0.619	0.059872
TC0600008632.hg.1	BCKDHB	-0.6212275	0.024301
TC0200015087.hg.1	SESTD1	-0.6242475	0.078749
TC0600014084.hg.1	HIST1H4I	-0.62729	0.032948
TC0100015711.hg.1	HIST2H2AB	-0.63107	0.027894
TC0100017358.hg.1	SLC30A10	-0.631445	0.040063
TC0600008319.hg.1	FAM83B	-0.63757	0.019857
TC0700011905.hg.1	BAIAP2L1	-0.6401625	0.053604
TC1100012229.hg.1	KDELC2	-0.6413525	0.028639
TC0200012754.hg.1	FAM161A	-0.64235	0.070844
TC0200015650.hg.1	FN1	-0.642975	0.057653
TC0600007301.hg.1	BTN3A2	-0.643595	0.0134
TC0600007282.hg.1	HIST1H2BF	-0.6442425	0.091951
TC1200007892.hg.1	ARHGEF25	-0.6452775	0.012696
TC2000007089.hg.1	TPX2	-0.6465225	0.019857
TC1000010758.hg.1	RTKN2	-0.64655	0.026376
TC0900011246.hg.1	ZNF883	-0.64673	0.090778
TC1700010560.hg.1	PLXDC1	-0.6474825	0.029223
TC0700009083.hg.1	AHCYL2	-0.65429	0.07611
TC2200007672.hg.1	GRAMD4	-0.656595	0.063597
TC0900009606.hg.1	BNC2	-0.6580725	0.061048

TC0200010421.hg.1	AOX1	-0.6602825	0.077675
:Unmapped00000732.hg	SLC37A4	-0.661065	0.070844
TC1600011375.hg.1	QPRT	-0.6633925	0.068526
TC0700013342.hg.1	CHN2	-0.6654175	0.030072
TC0X00006791.hg.1	PTCHD1	-0.6654625	0.082468
TC0600011138.hg.1	HIST1H1D	-0.666355	0.047927
TC0200013376.hg.1	EIF2AK3	-0.6663825	0.022541
TC1800006484.hg.1	NDC80	-0.666565	0.090778
TC0300007077.hg.1	ACVR2B	-0.66866	0.030104
TC1400009770.hg.1	ZDHHC22	-0.6698625	0.061141
TC0300011324.hg.1	FHIT	-0.670895	0.079699
TC0X00009107.hg.1	GEMIN8	-0.6780575	0.054722
TC0300008853.hg.1	TMEM108	-0.67977	0.009488
TC1300009984.hg.1	SLITRK5	-0.687275	0.023634
TC0600011224.hg.1	HIST1H2BL	-0.68838	0.037257
TC0200014785.hg.1	FIGN	-0.6893825	0.061033
TC0X00008017.hg.1	PLP1	-0.69166	0.08286
TC2100007872.hg.1	GRIK1	-0.6922025	0.092589
TC1900008012.hg.1	CATSPERG	-0.6937725	0.036649
TC1800008317.hg.1	ZNF521	-0.69379	0.013435
TC0400010242.hg.1	PPARGC1A	-0.6955275	0.038873
TC0X00011083.hg.1	TMEM185A	-0.69785	0.017943
TC1900007688.hg.1	CCNE1	-0.6995975	0.089615
TC0200015601.hg.1	KANSL1L	-0.7008075	0.062066
TC0300014073.hg.1	PLCH1	-0.7066325	0.011167
TC0400012473.hg.1	GPM6A	-0.7091075	0.084891
TC0900007904.hg.1	SYK	-0.711215	0.013133
TC1200010795.hg.1	RARG	-0.716465	0.036584
TC0100008779.hg.1	ST6GALNAC3	-0.716785	0.019885
TC1700011364.hg.1	BRIP1	-0.7214625	0.008043
TC1400010777.hg.1	TC2N	-0.7342875	0.028639
TC1800009029.hg.1	CYB5A	-0.7359325	0.017866
TC0200014845.hg.1	SPC25	-0.7360025	0.038773
TC1100007430.hg.1	GYLTL1B	-0.7398125	0.09616
TC0X00006891.hg.1	GK	-0.74031	0.039397
TC0500011157.hg.1	GCNT4	-0.740835	0.019857
TC1000009851.hg.1	FRMD4A	-0.741735	0.022541
TC0600011123.hg.1	HIST1H4B	-0.7418625	0.04756
TC0600011127.hg.1	HIST1H1C	-0.7450425	0.034278
TC0800012330.hg.1	NDUFAF6	-0.745605	0.042782
TC1600010532.hg.1	CDH8	-0.7462675	0.009488
TC1300006802.hg.1	BRCA2	-0.748595	0.008043
TC0600007375.hg.1	HIST1H3H	-0.751355	0.021503
TC1000010843.hg.1	PBLD	-0.75385	0.033907
TC0500012662.hg.1	ZBED8	-0.75501	0.068526
TC1200012741.hg.1	ANO2	-0.7587225	0.019561
TC1200010490.hg.1	NELL2	-0.75927	0.008043
TC1100012526.hg.1	USP2	-0.76691	0.048814
TC0700007426.hg.1	ADCY1	-0.7716925	0.014123
:Unmapped00000754.hg	FMN1	-0.7721725	0.017665

TC1600009545.hg.1	ABCC6	-0.7737525	0.009083
TC0400006639.hg.1	NSG1; D4S234E	-0.77496	0.01508
TC0100016431.hg.1	TNFSF4	-0.7750025	0.061048
TC0700006619.hg.1	SLC29A4	-0.7821825	0.013906
TC1000009714.hg.1	PRKCQ	-0.7865475	0.085207
TC1600009954.hg.1	MAPK3	-0.7881425	0.006068
TC1000008995.hg.1	ATRNL1	-0.7920075	0.035368
TC1900008946.hg.1	GALP	-0.795335	0.036992
;Unmapped00000626.hg	FMN1	-0.8010875	0.038026
TC1700012457.hg.1	AXIN2	-0.8048775	0.011167
TC1100011886.hg.1	RAB38	-0.810565	0.055828
TC0400008879.hg.1	GAB1	-0.8180225	0.040117
TC1100008553.hg.1	KCTD21-AS1	-0.8191725	0.034155
TC1500010160.hg.1	CTSH	-0.81979	0.025348
TC0600009710.hg.1	STX11	-0.82215	0.041278
TC0100009333.hg.1	GPSM2	-0.822295	0.014765
TC0900006438.hg.1	DOCK8	-0.82655	0.019885
TC0100009241.hg.1	S1PR1	-0.8277725	0.049675
TC1000007072.hg.1	APBB1IP	-0.82867	0.019885
TC0200016555.hg.1	TBR1	-0.82908	0.009488
TC0100014543.hg.1	DEPDC1	-0.831875	0.053738
TC0400009258.hg.1	PALLD	-0.8327325	0.021254
TC1200006647.hg.1	GNB3	-0.8337175	0.05059
TC0900009905.hg.1	CNTFR	-0.8349375	0.08467
TC0300012112.hg.1	B4GALT4	-0.840175	0.023634
TC0200014790.hg.1	GRB14	-0.840755	0.088127
TC1200011588.hg.1	USP44	-0.849695	0.009372
TC1400009684.hg.1	ALDH6A1	-0.85366	0.09271
TC1500010422.hg.1	IDH2	-0.85662	0.008237
TC0600012597.hg.1	EPHA7	-0.86599	0.028639
TC1800007052.hg.1	ASXL3	-0.86946	0.00672
TC0600008303.hg.1	LRRC1	-0.8702175	0.007953
TC0400009543.hg.1	TLR3	-0.874755	0.036649
TC0800011243.hg.1	FBXO43	-0.87494	0.043948
TC0200016073.hg.1	KCNJ13	-0.8829675	0.002555
TC0800009970.hg.1	PBK	-0.9007775	0.088857
TC0400012798.hg.1	ADGRL3	-0.9028775	0.060052
TC0100016218.hg.1	RGS5	-0.90433	0.060052
TC1500008794.hg.1	OCA2	-0.90957	0.063352
TC0500008454.hg.1	PRR16	-0.90989	0.042782
TC1000008875.hg.1	ADD3	-0.91225	0.039397
TC0X00011279.hg.1	TSPAN7	-0.9235	0.036059
TC1200011460.hg.1	KITLG	-0.923775	0.084891
TC1100010237.hg.1	ABCC8	-0.934985	0.029311
TC0400009860.hg.1	STX18	-0.9354225	0.008708
TC1200009985.hg.1	GSG1	-0.938585	0.008043
TC1000008271.hg.1	CDHR1	-0.9505275	0.026984
TC0400011916.hg.1	PCDH18	-0.9615175	0.047361
TC0800012322.hg.1	CA13	-0.97362	0.005816
TC1200011246.hg.1	PTPRR	-0.97829	0.050492

TC1400009617.hg.1	DPF3	-0.9976825	0.014765
TC0700008873.hg.1	CAV1	-1.009893	0.017376
TC0200015093.hg.1	ZNF385B	-1.013732	0.022805
TC1000012213.hg.1	FAM196A	-1.016845	0.057655
TC1100012794.hg.1	ARHGAP32	-1.01755	0.070844
TC1300008894.hg.1	RCBTB2	-1.031955	0.008694
TC0400007542.hg.1	KIT	-1.041875	0.009372
TC0X00006709.hg.1	NHS	-1.043988	0.008043
TC0600008038.hg.1	GUCA1A	-1.046247	0.013435
TC1700007408.hg.1	PIPOX	-1.059825	0.027265
TC1500008350.hg.1	SV2B	-1.06046	0.028639
TC1000011114.hg.1	KCNMA1	-1.072185	0.024914
TC1900011507.hg.1	ZSCAN5B	-1.083235	0.040396
TC1700011574.hg.1	ABCA8	-1.08391	0.003329
;Unmapped00000713.hg	FMN1	-1.11694	0.019857
TC0500010615.hg.1	SEPP1	-1.117025	0.034278
TC0400009037.hg.1	MND1	-1.142222	0.069087
TC1600007448.hg.1	CORO1A	-1.14618	0.020484
TC1800006571.hg.1	ARHGAP28	-1.151803	0.048814
TC0200016755.hg.1	NEUROD1	-1.16906	0.068526
TC0300011251.hg.1	ERC2	-1.18397	0.00672
TC0300008271.hg.1	BBX	-1.194727	0.011167
TC0100008496.hg.1	FGGY	-1.2180675	0.054536
TC0X00010428.hg.1	GLRA4	-1.239075	0.019885
TC0100011788.hg.1	MIXL1	-1.2484375	0.015902
TC0400011263.hg.1	SPARCL1	-1.288755	0.083707
;Unmapped00000559.hg	FMN1	-1.304358	0.015985
TC1700008757.hg.1	MAP2K6	-1.30536	0.009083
TC0200014728.hg.1	5-CD302; CD302; L	-1.339482	0.040117
TC1400007441.hg.1	AKAP5	-1.368015	0.003981
TC1000012579.hg.1	IT1; ARHGAP19-SLI	-1.371058	0.008995
TC1700010019.hg.1	MFAP4	-1.373882	0.024914
TC0300011852.hg.1	IMPG2	-1.416033	0.019885
TC0800008028.hg.1	PKIA	-1.475145	0.021679
TC0100017307.hg.1	USH2A	-1.502125	0.013395
TC1200008176.hg.1	LGR5	-1.504503	0.009083
TC0100016254.hg.1	RXRG	-1.5780325	0.035469
TC0500008470.hg.1	SNCAIP	-1.747532	0.033595
TC1700008673.hg.1	CACNG5	-1.814355	0.003243
TC0500009301.hg.1	GABRA6	-1.859058	0.015985
TC1400006562.hg.1	RPGRIP1	-2.01608	0.001682
TC1300009488.hg.1	DCT	-3.01387	0.006068

U87 NS

Affymetrix_ID	GeneSymbol	logFC (PEF/Sham)	adj.P.Val
TC2200008515.hg.1	BPIFC	1.1567775	0.004027064
TC1200010755.hg.1	KRT75	1.1315325	0.009877926
TC0500012392.hg.1	SPINK1	0.88713	0.031290592
TC0300011951.hg.1	MORC1	0.83622	0.069665424
TC0100015847.hg.1	SPRR2D	0.819075	0.005200956
TC0700009134.hg.1	CPA4	0.81749	0.01247524
TC0800008140.hg.1	ATP6V0D2	0.80054	0.011058322
TC0200016511.hg.1	IL1RN	0.7707775	0.005200956
TC1500007409.hg.1	GCNT3	0.751575	0.011726578
TC0800011646.hg.1	HAS2	0.729155	0.005200956
TC0700011876.hg.1	ASNS	0.7238675	0.005637494
TC0700009145.hg.1	MEST	0.7146725	0.011058322
TC0800008667.hg.1	NOV	0.7090225	0.004027064
TC0400007776.hg.1	FDCSP	0.661655	0.013312527
TC1800006733.hg.1	PRELID3A	0.6435375	0.005200956
TC1700011558.hg.1	SLC16A6	0.639685	0.011058322
TC0200013922.hg.1	IL36B	0.6345075	0.008102
TC2000008971.hg.1	GDF5	0.6232775	0.0114496
TC0800012386.hg.1	PINX1; MIR1322	0.6196325	0.009877926
TC0500012870.hg.1	STC2	0.61875	0.004027064
TC1000007176.hg.1	MAP3K8	0.610515	0.067443164
TC2200007204.hg.1	HMOX1	0.60623	0.0114496
TC0900010971.hg.1	TBC1D2	0.594405	0.025547592
TC2200007523.hg.1	SERHL	0.593735	0.043671166
TC1100012717.hg.1	PUS3	0.5899625	0.019594536
TC0200013899.hg.1	ZC3H8	0.5717025	0.026473153
TC0100009394.hg.1	CD53	0.567535	0.021228758
TC0900009861.hg.1	NOL6	0.562815	0.0114496
TC0300008550.hg.1	CSTA	0.54724	0.066496062
TC0700009411.hg.1	MGAM2	0.54379	0.017257589
TC1100013202.hg.1	ORAOV1	0.53871	0.035844342
TC1900006863.hg.1	LRRC8E	0.53866	0.046330913
TC0200016501.hg.1	IL1RL1	0.5358525	0.0114496
TC0X00008392.hg.1	UTP14A	0.5293425	0.011058322
TC0900007667.hg.1	PSAT1	0.5266675	0.008102
TC0700010582.hg.1	HIBADH	0.526495	0.066245496
TC0500008632.hg.1	SLC22A5	0.5218825	0.0512073
TC0700011010.hg.1	SUN3	0.5121825	0.030721619
TC0500012760.hg.1	SLIT3	0.5106325	0.067443164
TC1000011992.hg.1	SFXN4	0.5104925	0.021865032
TC0100016006.hg.1	GPATCH4	0.50834	0.008102
TC1900010836.hg.1	PSG4	0.500735	0.025547592
Unmapped00000292.hg	TGM4	0.4998025	0.09268633
TC1600011347.hg.1	ALG1; NAGPA-AS1	0.497595	0.076707853
TC0700013579.hg.1	SEMA3A	0.4948275	0.092203455
TC1800009242.hg.1	SERPINB2	0.4896025	0.021865032
TC1100007884.hg.1	SLC3A2	0.487385	0.010955303
TC1000009169.hg.1	ACADSB	0.48605	0.065232968

TC0200014764.hg.1	DPP4	0.485315	0.040810003
TC1600011186.hg.1	SLC7A5	0.4850525	0.011058322
TC1400010195.hg.1	WARS	0.48459	0.01247524
TC0100018492.hg.1	SPRR2A	0.4813425	0.019594536
TC1000007976.hg.1	CHST3	0.4744125	0.017263356
TC0400010141.hg.1	LDB2	0.4673125	0.092638529
TC1200008606.hg.1	UTP20	0.465655	0.062798851
TC1500008304.hg.1	GDPGP1	0.4645875	0.046873561
TC1500008202.hg.1	AGBL1	0.4645	0.046330913
TC0100014210.hg.1	COA7	0.4626325	0.034996871
TC0700012971.hg.1	ZNF425	0.46113	0.085962898
TC1600009677.hg.1	CRYM	0.4592375	0.033167433
TC1100013012.hg.1	STX3	0.456645	0.012075777
TC0400012989.hg.1	TMEM192	0.4555625	0.097429958
TC0400012941.hg.1	THAP9-AS1	0.45519	0.049561569
TC0400012874.hg.1	CYP4V2	0.454755	0.01247524
TC1700012460.hg.1	ABCA5	0.453065	0.091684275
TC0700009240.hg.1	AGBL3	0.45297	0.089179173
TC0100010713.hg.1	PAPPA2	0.45211	0.092548463
TC0200016344.hg.1	MTERF4	0.4510075	0.013312527
TC0100014502.hg.1	WDR78	0.4489125	0.094919693
TC0400007285.hg.1	LIAS	0.4487075	0.085583092
TC2200006832.hg.1	RAB36	0.44585	0.02238493
TC0700013067.hg.1	SMARCD3	0.442155	0.066567516
TC2000009259.hg.1	NCOA5	0.43869	0.024152102
TC0900010792.hg.1	NOL8	0.438295	0.046873561
TC1700006585.hg.1	CTNS	0.43482	0.049010227
TC0X00007107.hg.1	KRBOX4	0.43389	0.022248627
TC0700013441.hg.1	IFRD1	0.4321075	0.011058322
TC1300007870.hg.1	ITGBL1	0.43065	0.052112489
TC0400009137.hg.1	FNIP2	0.4247025	0.073252833
TC0700012148.hg.1	SRPK2	0.42385	0.046873561
TC0900010886.hg.1	PTCH1	0.4228775	0.03435774
TC0600013542.hg.1	ULBP3	0.4226575	0.043671166
TC0500007138.hg.1	IL7R	0.42179	0.031580265
TC1000008665.hg.1	C10orf2	0.421485	0.033221637
TC0X00010652.hg.1	UPF3B	0.4211975	0.020215418
TC0300007015.hg.1	ARPP21	0.4205225	0.026457334
TC0100013602.hg.1	TMEM234	0.4189625	0.033273608
TC0100009204.hg.1	TRMT13	0.415525	0.037818217
TC0800006913.hg.1	SH2D4A	0.4136975	0.04800286
TC0100006991.hg.1	RSC1A1; DDI2	0.4131575	0.033167433
TC0300008336.hg.1	C3orf52	0.4111725	0.034996871
TC0900007452.hg.1	ZNF658	0.41066	0.094396878
TC0400007923.hg.1	MRPL1	0.410005	0.034996871
TC1500006788.hg.1	GREM1	0.408665	0.040810003
TC1000008770.hg.1	PDCD11	0.4082675	0.034332503
TC0200008949.hg.1	IL36RN	0.406585	0.017759987
TC0200012642.hg.1	MTIF2	0.40577	0.021850769
TC1200008792.hg.1	UNG	0.40494	0.0512073

TC1600010498.hg.1	SLC38A7	0.4046575	0.040005001
TC0500012568.hg.1	GEMIN5	0.4029825	0.054474791
TC0900008318.hg.1	TMEM38B	0.4026825	0.027911185
TC1200008748.hg.1	PWP1	0.4025475	0.061960415
TC2000006541.hg.1	ITPA	0.40153	0.053535836
TC1700009150.hg.1	BAIAP2	0.400905	0.038822528
TC0100009621.hg.1	PHGDH	0.4009025	0.025111113
TC1900011978.hg.1	ZNF296	0.40042	0.059928963
TC0200013137.hg.1	GCFC2	0.40038	0.052350227
TC0400012396.hg.1	AADAT	0.399945	0.0512073
TC0400012934.hg.1	SDAD1	0.3981675	0.092203455
TC1500010723.hg.1	CHAC1	0.39776	0.099093194
TC1500009639.hg.1	ICE2	0.3966625	0.072373494
TC0100015851.hg.1	SPRR2E	0.3961825	0.0512073
TC1100012209.hg.1	ALKBH8	0.393825	0.033225252
TC1500007355.hg.1	AQP9	0.3937625	0.014112572
TC1400008583.hg.1	CCNB1IP1; SNORD126	0.3933475	0.100575866
TC1900011775.hg.1	GRWD1	0.39292	0.05919168
TC2200007406.hg.1	ATF4	0.392545	0.049283756
TC0300014095.hg.1	TM4SF19	0.3919675	0.094396878
TC0400012179.hg.1	PLRG1	0.391875	0.059776557
TC0400010258.hg.1	LGI2	0.391505	0.076707853
TC0400008091.hg.1	SPP1	0.3912075	0.049240832
TC2200008932.hg.1	KIAA1644	0.3904925	0.042123027
TC1200008729.hg.1	POLR3B	0.3899125	0.0512073
TC1900008564.hg.1	ATF5; MIR4751	0.389675	0.064424936
TC0900008380.hg.1	FAM206A	0.3875875	0.065460505
TC0300012186.hg.1	CCDC58	0.3857775	0.055553424
TC0200007115.hg.1	CLIP4	0.384845	0.090665143
TC0300006465.hg.1	LRRN1	0.384825	0.094970292
TC0200015514.hg.1	GPR1	0.3841525	0.092004163
TC0500007690.hg.1	SERF1B; SERF1A	0.3826175	0.076769831
TC0500007698.hg.1	SERF1A	0.3826175	0.076769831
TC0700012596.hg.1	TMEM209	0.3825175	0.030139204
TC0300011009.hg.1	CDC25A	0.381665	0.033225252
TC2000008318.hg.1	FERMT1	0.38079	0.034996871
TC0700013583.hg.1	MTERF1	0.3802475	0.067361676
TC0500007542.hg.1	NDUFAF2	0.3800375	0.069665424
TC0100013581.hg.1	SPOCD1	0.37947	0.073798434
TC0500013153.hg.1	MTRR	0.3790825	0.021865032
TC1100013075.hg.1	DDIAS	0.377945	0.0512073
TC0200010480.hg.1	NOP58	0.377465	0.019594536
TC0100017267.hg.1	ANGEL2	0.3774275	0.0512073
TC0500010869.hg.1	ELOVL7	0.3760525	0.043671166
TC1100012811.hg.1	NFRKB	0.37522	0.063760834
TC0200006891.hg.1	RHOB	0.3751325	0.054591271
TC0100009900.hg.1	CIART	0.372915	0.043671166
TC0200016702.hg.1	POLR1A	0.3719425	0.042123027
TC1600010809.hg.1	TXNL4B	0.37165	0.059776557
TC0100012921.hg.1	DHRS3; MIR6730	0.37106	0.032071873

TC0300012195.hg.1	HSPBAP1	0.3708	0.053681113
TC0800008487.hg.1	RIMS2	0.36995	0.055553424
TC2000008268.hg.1	SLC23A2	0.3682825	0.072373494
TC1900008036.hg.1	MRPS12	0.368275	0.064399995
TC0500012302.hg.1	GNPDA1	0.3674575	0.0512073
TC1900007418.hg.1	ARMC6	0.36723	0.034996871
TC1400007975.hg.1	C14orf159	0.3665075	0.067443164
TC1600011440.hg.1	OSGIN1	0.3664175	0.085204682
TC1000007847.hg.1	MYPN	0.3651675	0.03994713
TC2200006636.hg.1	GCR8; MIR1306; MIR36	0.3643575	0.024152102
TC0900011370.hg.1	BRINP1	0.3639825	0.087508437
TC1900008692.hg.1	ZNF480	0.36192	0.033167433
TC1000011904.hg.1	ABLIM1	0.3608825	0.053535836
TC0500010779.hg.1	SLC38A9	0.3589775	0.059587735
TC0200016772.hg.1	NHEJ1	0.3588625	0.027911185
SUnmapped00000594.hg	GREM1	0.3586175	0.0512073
TC0900009645.hg.1	HAUS6	0.357725	0.053535836
TC2100007822.hg.1	ADAMTS5	0.3575875	0.021865032
TC1200012714.hg.1	HSPB8	0.3575625	0.040254667
TC0500009123.hg.1	SLC36A1	0.35691	0.093282551
TC0X00010211.hg.1	CHM	0.3562975	0.06328314
TC1200012650.hg.1	MARS; MIR6758	0.356245	0.02654718
TC1800009222.hg.1	RNF125	0.3561975	0.084675912
TC1100006976.hg.1	MYOD1	0.35551	0.09268633
TC0800007985.hg.1	GDAP1	0.3540525	0.060671965
TC0500011529.hg.1	RIOK2	0.35063	0.0512073
TC0100010872.hg.1	SMG7	0.350605	0.061588776
TC1500009217.hg.1	LCMT2	0.3501475	0.036955529
TC1200008126.hg.1	CPSF6	0.3501075	0.052434394
TC0500011095.hg.1	MRPS27	0.3497175	0.033221637
TC0900009259.hg.1	UAP1L1	0.34917	0.0512073
TC0300014021.hg.1	CPOX	0.3487725	0.092203455
TC0500012145.hg.1	KLHL3	0.34829	0.092203455
TC0700009692.hg.1	SLC4A2	0.346065	0.055553424
TC1000010411.hg.1	ZNF33B	0.345245	0.099272227
TC1100007826.hg.1	BEST1	0.3441875	0.052143138
TC0200008806.hg.1	LIMS1	0.3439925	0.041963274
TC1000007081.hg.1	PDSS1	0.34384	0.043587405
TC1900008180.hg.1	ZNF526	0.3436225	0.024899557
TC1500006977.hg.1	VPS18	0.3420575	0.074271886
TC0500013259.hg.1	MFAP3	0.3415	0.049095383
TC1500009597.hg.1	MYO1E	0.340895	0.043804042
TC0100009373.hg.1	SLC6A17	0.340875	0.054474791
TC1600010603.hg.1	RRAD	0.34057	0.027911185
TC0800011887.hg.1	ST3GAL1	0.3402275	0.039714129
TC1800008779.hg.1	NARS	0.33964	0.071470684
TC0100016364.hg.1	SCYL3	0.3396225	0.083270988
TC2000009198.hg.1	ADA	0.3395825	0.084213367
TC0X00010409.hg.1	BEX2	0.33957	0.092203455
TC0600012752.hg.1	PDSS2	0.33926	0.078710038

TC1600009514.hg.1	RRN3	0.339105	0.069975781
TC1100011930.hg.1	CHORDC1	0.3383525	0.083330937
TC0X00010880.hg.1	MOSPD1	0.338095	0.043671166
TC1200010516.hg.1	SLC38A1	0.337885	0.077859436
TC0200008259.hg.1	MAT2A	0.33752	0.072373494
TC0X00007356.hg.1	GNL3L	0.3369975	0.094919693
TC0100008815.hg.1	IFI44L	0.3366775	0.065232968
TC2100008061.hg.1	SETD4	0.3363875	0.069207837
TC1100012984.hg.1	NAT10	0.33553	0.052434394
TC0200013894.hg.1	ANAPC1	0.3334225	0.072373494
TC0900009779.hg.1	C9orf72	0.3333025	0.099272227
TC1600008190.hg.1	SLC7A6	0.333035	0.031751474
TC0X00007923.hg.1	CSTF2	0.3327325	0.069286354
TC0200016651.hg.1	CYP1B1	0.33166	0.059587735
TC0400009791.hg.1	NOP14	0.3315975	0.073895931
TC0600014136.hg.1	ADGRF4	0.3312225	0.082427867
TC0400008335.hg.1	AIMP1	0.330075	0.027898097
TC0300009815.hg.1	LPP	0.3298575	0.055553424
TC0800011683.hg.1	FBXO32	0.329265	0.046330913
TC2000006642.hg.1	BMP2	0.328795	0.099876826
TC0100008121.hg.1	TOE1	0.3283525	0.046873561
TC0800010458.hg.1	TCEA1	0.3282175	0.037818217
TC0700013396.hg.1	CLDN12	0.32796	0.067443164
TC0800008715.hg.1	ZHX2	0.3265175	0.099876826
TC0200016224.hg.1	PER2	0.32648	0.094919693
TC0500012937.hg.1	NOP16	0.32591	0.069665424
TC1000009002.hg.1	PNLIPRP3	0.3256425	0.035844342
TC1900008989.hg.1	ZNF460	0.325375	0.043671166
TC1700011917.hg.1	USP36	0.3252825	0.056814452
TC0200014049.hg.1	TMEM185B	0.325165	0.072373494
TC0100013629.hg.1	YARS	0.3248375	0.049561569
TC1400008705.hg.1	SLC7A8	0.3245275	0.025740583
TC2000006518.hg.1	D56; SNORA51; SNORD	0.32443	0.0512073
TC1600011368.hg.1	LAT	0.32318	0.072373494
TC0300006489.hg.1	ARL8B	0.322245	0.092776123
TC0100013184.hg.1	MUL1	0.322205	0.061960415
TC1100007050.hg.1	PRMT3	0.3215275	0.069665424
TC0200014907.hg.1	SLC25A12	0.32096	0.067361676
TC0100016263.hg.1	TMCO1	0.3207375	0.092203455
TC0200013641.hg.1	CHST10	0.3201475	0.072373494
TC0900011846.hg.1	REXO4	0.3200825	0.070219298
TC1700006774.hg.1	CYB5D1	0.3200275	0.051196291
TC0500009706.hg.1	SQSTM1	0.3199525	0.034996871
TC1500010887.hg.1	CLN6	0.319785	0.057870475
TC2200008447.hg.1	PES1	0.3195075	0.034996871
TC0100017118.hg.1	YOD1	0.319055	0.087810386
;Unmapped00000090.hg	REXO4	0.3186325	0.046873561
TC1100006831.hg.1	ADM	0.3184475	0.053535836
TC1000011130.hg.1	DLG5	0.31818	0.057278562
TC0800011844.hg.1	ADCY8	0.317575	0.072373494

TC1100011022.hg.1	VPS37C	0.3173	0.062084018
TC2000008439.hg.1	ESF1	0.316955	0.0512073
TC0200007886.hg.1	PNO1	0.3165075	0.071969365
TC1600009966.hg.1	DCTPP1	0.316495	0.092203455
TC0100009344.hg.1	SARS	0.316405	0.070749101
TC1600010734.hg.1	NOB1	0.3159625	0.060248261
TC1900008323.hg.1	CD3EAP	0.3154125	0.094771752
[illegible]Unmapped00000571.hg	GREM1	0.31485	0.05690032
TC0100014964.hg.1	DNTTIP2	0.31458	0.0512073
TC1200008028.hg.1	MSRB3	0.3142225	0.0512073
TC1700006464.hg.1	FAM57A	0.3142	0.099272227
TC0200007111.hg.1	WDR43; SNORD53	0.3136275	0.082880292
TC0700008743.hg.1	PIK3CG	0.3128525	0.058568607
TC0300008352.hg.1	GTPBP8	0.3126525	0.087810386
TC1900011730.hg.1	TIMM50	0.3123775	0.087590101
TC2100007821.hg.1	ADAMTS1	0.3106425	0.046649477
TC1700009590.hg.1	KIAA0753	0.31002	0.062798851
TC2100008499.hg.1	SMIM11A	0.3092825	0.056626913
TC1700012342.hg.1	DHX33	0.30897	0.0512073
TC2100006460.hg.1	SMIM11A	0.3085575	0.057802668
TC0800007870.hg.1	CSPP1	0.3085375	0.05528003
TC1100011107.hg.1	WDR74	0.307175	0.043671166
TC0100008080.hg.1	DPH2	0.306545	0.068658978
TC0600006926.hg.1	PAK1IP1	0.306205	0.046873561
TC0600012671.hg.1	ASCC3	0.306155	0.091684275
TC1700011208.hg.1	TRIM25; MIR3614	0.30583	0.0512073
TC0700010925.hg.1	DDX56	0.305645	0.051509395
TC1200010281.hg.1	FAM60A	0.305075	0.065232968
TC0800007978.hg.1	LY96	0.30422	0.073798434
TC0300006687.hg.1	CCDC174	0.3041725	0.072373494
TC0600012628.hg.1	NDUFAF4	0.303865	0.073798434
TC0100014918.hg.1	SETSIP	0.3033075	0.035493258
TC1700008945.hg.1	SPHK1	0.3031675	0.092004163
TC0700012602.hg.1	CEP41	0.301575	0.069665424
TC1600008979.hg.1	NARFL	0.30094	0.099349948
TC1200011591.hg.1	NTN4	0.3006925	0.072373494
TC0100009580.hg.1	WDR3	0.29949	0.082427867
TC1300009712.hg.1	EFNB2	0.299005	0.068777704
TC0400012854.hg.1	TMEM144	0.298065	0.05495911
[illegible]Unmapped00000078.hg	SLC16A1	0.29761	0.051988173
TC0700010692.hg.1	NT5C3A	0.2975575	0.083270988
TC0600007447.hg.1	OR2J3	0.297205	0.085583092
TC1700007444.hg.1	NSRP1; MIR423	0.29711	0.095424527
TC0700009980.hg.1	PRKAR1B	0.2969375	0.0512073
TC1900007763.hg.1	GPATCH1	0.296825	0.092004163
TC0400012670.hg.1	FAT1	0.296725	0.063760834
TC0100011382.hg.1	DYRK3	0.29517	0.073500874
TC0600007250.hg.1	SLC17A4	0.2943825	0.094919693
TC2100006442.hg.1	SIK1	0.2939975	0.070219298
TC0300008964.hg.1	MRPS22	0.29271	0.074271886

TC2100008384.hg.1	UBE2G2	0.2927025	0.078066478
TC1000007889.hg.1	SUPV3L1	0.291935	0.090665143
TC1700011233.hg.1	MRPS23	0.291205	0.076769831
TC1900009766.hg.1	TRMT1	0.2907575	0.055553424
TC0300010769.hg.1	GORASP1	0.290015	0.068605755
TC1900008993.hg.1	ZNF304	0.2895375	0.071844857
TC1200008703.hg.1	C12orf45	0.2893125	0.071969365
TC0400008318.hg.1	TET2	0.28913	0.0512073
TC0900011840.hg.1	MED22	0.28898	0.09888254
TC1100011101.hg.1	NXF1	0.2889025	0.050951887
TC0800012405.hg.1	FUT10	0.2887425	0.065753633
TC1000012491.hg.1	ENTPD7	0.288285	0.059599562
TC0300006627.hg.1	TSEN2	0.2877675	0.041776133
TC0700009834.hg.1	.OC389602; AC021218.	0.2875725	0.098341085
TC0700008073.hg.1	POR; MIR4651	0.28706	0.092203455
TC1400009690.hg.1	ABCD4	0.2869275	0.087590101
TC1000008713.hg.1	NOLC1	0.286265	0.089329888
TC1700012217.hg.1	TMEM199; MIR4723	0.2857375	0.059776557
TC1200012003.hg.1	RBM19	0.2856975	0.066567516
TC0X00009537.hg.1	ZNF674	0.285645	0.072373494
TC1000012582.hg.1	RRP12	0.28469	0.059928963
TC0300013471.hg.1	BCL6	0.2840525	0.0512073
TC0700012122.hg.1	DNAJC2	0.2835275	0.076769831
TC0400011130.hg.1	PAQR3	0.28318	0.045606855
TC0500009059.hg.1	GRPEL2	0.282675	0.051196291
TC0800009301.hg.1	C8orf33	0.2826475	0.067091848
TC1200012804.hg.1	PRIM1	0.2817075	0.094919693
TC0700009488.hg.1	CASP2	0.2806175	0.065232968
TC1100011192.hg.1	ATG2A	0.280355	0.076769831
TC0700012760.hg.1	ZC3HAV1L	0.279945	0.099093194
TC1600010599.hg.1	NAE1	0.27984	0.074271886
TC2000010006.hg.1	EIF6	0.279675	0.0512073
TC0600014318.hg.1	FAM46A	0.2795525	0.072373494
TC0100014988.hg.1	F3	0.2791525	0.090253157
TC0200011726.hg.1	NOL10	0.2791425	0.078710038
TC0700006913.hg.1	GPNMB	0.2786725	0.051196291
TC1000008423.hg.1	RPP30	0.27813	0.069207837
TC2100008297.hg.1	SIK1	0.2777725	0.095111596
TC0700013119.hg.1	XRCC2	0.27744	0.081621662
TC1000010818.hg.1	CTNNA3	0.277345	0.099626159
TC0200016507.hg.1	LIMS3L; LIMS3	0.2772725	0.087590101
TC0900007488.hg.1	PIP5K1B	0.27722	0.094919693
TC1700007585.hg.1	SLFN5	0.2768375	0.092004163
TC0100018567.hg.1	RBM34	0.2754025	0.067443164
TC1600010450.hg.1	CIAPIN1	0.275335	0.065232968
TC0600009142.hg.1	RPF2	0.274875	0.083674222
TC0300009625.hg.1	TTC14	0.274705	0.100493558
TC1600009420.hg.1	RSL1D1	0.2742475	0.062798851
TC1100013165.hg.1	SLC43A3	0.273295	0.056626913
TC0300010388.hg.1	MRPS25	0.273175	0.095026515

TC0100011619.hg.1	RRP15	0.27291	0.092376425
;Unmapped00000486.hg	MLXIP	0.2718125	0.092429672
TC0900007816.hg.1	CTSL	0.2716325	0.069975781
TC1700010798.hg.1	DUSP3	0.27146	0.097429958
TC0100015817.hg.1	THEM4	0.271335	0.059928963
TC0X00007195.hg.1	RBM3	0.2711225	0.069339163
TC0500008919.hg.1	RNF14	0.2707525	0.092376425
TC0200016603.hg.1	LRRFIP1	0.2699775	0.099272227
TC0300007259.hg.1	CCRL2	0.269125	0.092203455
TC0600013361.hg.1	CITED2	0.2669125	0.068605755
TC0X00009952.hg.1	AWAT2	0.2664275	0.097429958
TC0200007195.hg.1	TTC27; MIR4765	0.2656725	0.094919693
TC0600007711.hg.1	ITPR3	0.2646325	0.078490128
TC0300009694.hg.1	ABCF3	0.2634875	0.085204682
TC0800009872.hg.1	TNFRSF10D	0.2631575	0.076298922
TC0500007457.hg.1	SETD9	0.26262	0.07498156
TC0800007245.hg.1	MAK16	0.262355	0.094919693
TC0200015976.hg.1	SP110	0.2616125	0.094919693
TC0700010182.hg.1	PMS2	0.2611125	0.069207837
TC0300012315.hg.1	TPRA1	0.2609575	0.087384918
;Unmapped00000461.hg	ZNF35	0.25698	0.092203455
TC1200011301.hg.1	KRR1	0.2569075	0.085962898
TC1300006965.hg.1	WBP4	0.2560575	0.098341085
TC0300013829.hg.1	RBM5	0.2552275	0.084868225
TC1200008370.hg.1	C12orf29	0.25504	0.100076993
TC0500012163.hg.1	CDC23	0.2545025	0.092004163
TC0800006933.hg.1	ATP6V1B2	0.254305	0.095208762
TC0200012066.hg.1	SLC5A6	0.25411	0.094919693
TC0100010284.hg.1	PEA15	0.2537875	0.08784533
TC0700010435.hg.1	CDCA7L	0.2534225	0.067091848
TC2000010022.hg.1	ZNFX1	0.25298	0.099876826
TC1200012591.hg.1	EMG1	0.2527	0.092203455
TC0700007334.hg.1	MRPL32	0.25226	0.062084018
TC0800011372.hg.1	SLC25A32	0.2520175	0.082427867
TC1200008116.hg.1	MDM2	0.2507125	0.071969365
TC0100010102.hg.1	FLAD1	0.250395	0.077218737
TC0200010567.hg.1	EEF1B2; SNORA41	0.24958	0.069665424
TC0500007088.hg.1	TARS	0.247895	0.067443164
TC1600010752.hg.1	AARS	0.2468475	0.087398953
TC1100008797.hg.1	PANX1	0.2453825	0.069665424
TC1900011650.hg.1	ARHGEF18	0.24258	0.090253157
TC1700010501.hg.1	DDX52	0.2394525	0.09268633
TC0100010514.hg.1	MPZL1	0.23945	0.087590101
TC0200014597.hg.1	RND3	0.2389875	0.087810386
TC1700009451.hg.1	CLUH	0.23882	0.080460097
TC1700006466.hg.1	RNMTL1	0.2387075	0.092004163
TC1000007462.hg.1	ZNF22	0.2377275	0.099876826
TC1300008533.hg.1	HSPH1	0.236215	0.098341085
TC0500008864.hg.1	WDR55	0.2357375	0.095873626
TC1200008683.hg.1	CHST11	0.234745	0.071622008

TC1400009576.hg.1	2BP-COX16; COX16; SYN	0.2342575	0.091684275
TC1400008684.hg.1	PRMT5	0.2339925	0.092203455
TC0600013757.hg.1	SOD2	0.2330175	0.067443164
TC2100006852.hg.1	USP16	0.2321425	0.092776123
TC1700008498.hg.1	PPM1D	0.230865	0.09268633
TC0200011171.hg.1	SH3BP4	0.22828	0.095766335
TC0200015349.hg.1	HSPD1	0.2269825	0.072547477
TC1900008924.hg.1	U2AF2	0.2264975	0.08475445
TC0900012168.hg.1	MRRF	0.2250775	0.085583092
TC0300012728.hg.1	PLSCR1	0.22463	0.100076993
TC1200012161.hg.1	TRIAP1	0.2242425	0.094919693
TC1700009189.hg.1	HGS	0.2228025	0.092429672
TC0100015715.hg.1	SF3B4	0.2217625	0.092203455
TC2000007283.hg.1	SRC	0.21928	0.081470333
TC1600006995.hg.1	BFAR	0.2133925	0.100060779
TC1100011035.hg.1	CPSF7	0.2133475	0.100060779
TC1700012262.hg.1	PSME3	0.2132275	0.092203455
TC0900008484.hg.1	PRPF4	0.21252	0.090665143
TC0100018435.hg.1	EBNA1BP2; MIR6733	0.21012	0.092203455
TC0600014276.hg.1	HLA-DMB	-0.225435	0.094396878
TC0500009622.hg.1	GRK6	-0.22718	0.092203455
TC1600006610.hg.1	CCNF	-0.2283825	0.094919693
TC0300013598.hg.1	LRRC15	-0.228915	0.092203455
TC0700010321.hg.1	ETV1	-0.229145	0.076298922
TC1100013022.hg.1	FADS2	-0.232355	0.070219298
TC1100012976.hg.1	NUCB2	-0.23361	0.092638529
TC0300011173.hg.1	NT5DC2	-0.23388	0.087896495
TC0300012145.hg.1	FSTL1; MIR198	-0.2367625	0.08244143
TC0X00007170.hg.1	SPACA5B	-0.2404175	0.092203455
TC0900011631.hg.1	GOLGA2	-0.240485	0.094945237
TC1100011514.hg.1	DHCR7	-0.241065	0.081957036
TC1300008511.hg.1	KATNAL1	-0.2434875	0.094919693
TC0800010002.hg.1	DUSP4	-0.24427	0.092203455
TC0600009459.hg.1	ENPP1	-0.24806	0.065232968
TC2000006464.hg.1	FAM110A	-0.2484975	0.095424527
TC1500009522.hg.1	MNS1	-0.250135	0.066567516
TC1600008498.hg.1	VAT1L	-0.251785	0.06739708
TC1500006951.hg.1	KNSTRN	-0.25252	0.072628767
TC0600007301.hg.1	BTN3A2	-0.2527425	0.080119392
TC0600007700.hg.1	KIFC1	-0.2537025	0.076707853
TC0X00009044.hg.1	GPR143	-0.2543075	0.095111596
TC2000009058.hg.1	TGM2	-0.254875	0.056626913
TC1200012833.hg.1	PAH	-0.255685	0.092004163
TC0900009437.hg.1	AK3	-0.2564025	0.056814452
TC2200006675.hg.1	SERPIND1	-0.257235	0.092203455
TC0400011144.hg.1	ANTXR2	-0.258135	0.086655659
TC0900012154.hg.1	TMEFF1	-0.25828	0.100076993
TC0700007198.hg.1	ANLN	-0.25845	0.062084018
TC1500010018.hg.1	SEMA7A	-0.2591775	0.062653524
TC1700007042.hg.1	MPRIP	-0.25968	0.094005923

TC1200009621.hg.1	FOXM1	-0.260985	0.065232968
TC0700012968.hg.1	PDIA4	-0.2610425	0.076707853
TC1900006711.hg.1	TNFAIP8L1	-0.262	0.080119392
TC0600013280.hg.1	MAP3K5	-0.2631325	0.099876826
TC1100012516.hg.1	CCDC153	-0.2633925	0.065232968
TC0100016633.hg.1	RGS8	-0.266145	0.072517654
TC1500007695.hg.1	PAQR5	-0.2669275	0.050116522
TC1900009651.hg.1	YIPF2	-0.267085	0.072373494
TC2000009550.hg.1	AURKA	-0.26712	0.0512073
TC0100010381.hg.1	UAP1	-0.2716925	0.062761
TC1500007185.hg.1	FGF7	-0.2718325	0.072373494
TC1500007967.hg.1	DNAJA4	-0.27249	0.083270988
TC0100009067.hg.1	FNBP1L	-0.27303	0.085996293
TC1900011270.hg.1	HAS1	-0.273315	0.092376425
TC0100017066.hg.1	KLHDC8A	-0.2737425	0.087810386
TC0900008740.hg.1	OLFML2A	-0.274275	0.092203455
TC1200009136.hg.1	CABP1	-0.277285	0.091138941
TC0800006760.hg.1	FDFT1	-0.2775625	0.060248261
TC1000007748.hg.1	CDK1	-0.2777825	0.062084018
TC1200008873.hg.1	SH2B3	-0.2786375	0.076298922
TC0700007054.hg.1	PRR15	-0.2792225	0.072373494
TC0100010389.hg.1	HSD17B7	-0.2802025	0.081627833
TC1100010456.hg.1	PAX6	-0.2802875	0.09268633
TC0900008969.hg.1	ABL1	-0.28065	0.094406062
TC1000009536.hg.1	IDI1	-0.2806525	0.0512073
TC0200012452.hg.1	SIX2	-0.2808875	0.094005923
TC1200009759.hg.1	CDCA3	-0.2828825	0.067443164
TC0600011945.hg.1	RCAN2	-0.2831625	0.092203455
TC1000008891.hg.1	DUSP5	-0.283575	0.081494626
TC1000007883.hg.1	SRGN	-0.2836075	0.043671166
TC2200007662.hg.1	GTSE1	-0.28417	0.053535836
TC0400012102.hg.1	LRBA	-0.28493	0.060248261
TC0800010275.hg.1	PLAT	-0.285825	0.064787215
TC1200007147.hg.1	ARNTL2	-0.2862875	0.062084018
TC0X00009386.hg.1	SRPX	-0.2866075	0.043671166
TC1700010790.hg.1	ETV4	-0.286675	0.08475445
TC0500013244.hg.1	PCDHB16	-0.2867675	0.0512073
TC0300014063.hg.1	ANKUB1	-0.28811	0.070219298
TC0700008358.hg.1	COL1A2	-0.2882375	0.069665424
TC1400010261.hg.1	MOK	-0.2887125	0.09429802
TC0900009127.hg.1	COL5A1	-0.289425	0.072373494
TC0500007804.hg.1	HMGCR	-0.29009	0.072791484
TC0X00007267.hg.1	MAGED1	-0.2904825	0.095424527
TC0600013186.hg.1	CTGF	-0.29255	0.092203455
TC0900008675.hg.1	OR1L6	-0.292765	0.083456849
TC0500008620.hg.1	CSF2	-0.29339	0.092203455
TC1500010715.hg.1	BUB1B	-0.29367	0.062084018
TC0400012645.hg.1	SORBS2	-0.2969425	0.100388467
TC1600010561.hg.1	CDH11	-0.29745	0.066567516
TC1000009276.hg.1	DOCK1	-0.2979775	0.078160506

TC1600011508.hg.1	CDR2	-0.29877	0.085368395
TC0X00010670.hg.1	TMEM255A	-0.298825	0.074271886
TC2000008614.hg.1	NKX2-2	-0.299145	0.094919693
TC0600007511.hg.1	TRIM40	-0.29974	0.09268633
TC1700009476.hg.1	OR3A1	-0.2997425	0.095424527
TC0100008057.hg.1	CDC20	-0.3004925	0.0512073
TC0X00007191.hg.1	EBP	-0.300525	0.035844342
TC1700010618.hg.1	TOP2A	-0.3007225	0.046873561
TC0300013599.hg.1	GP5	-0.3014725	0.087590101
TC0400011015.hg.1	CXCL3	-0.3015475	0.0512073
TC0100011022.hg.1	RGS2	-0.301605	0.09429802
TC0400009221.hg.1	MSMO1	-0.303265	0.061960415
TC0100008101.hg.1	KIF2C	-0.3032925	0.085204682
TC0100013169.hg.1	PLA2G2C	-0.3034975	0.096017879
TC1900011770.hg.1	C5AR1	-0.304505	0.072373494
TC1200007640.hg.1	SLC4A8	-0.3047225	0.080119392
TC0900006527.hg.1	CDC37L1	-0.30503	0.092203455
TC0400012432.hg.1	HMGB2	-0.305405	0.072464434
TC0600009381.hg.1	CENPW	-0.3061625	0.065232968
TC0500008880.hg.1	PCDHB7	-0.3070175	0.099876826
TC1200010415.hg.1	SLC2A13	-0.3077775	0.092004163
TC0X00008724.hg.1	HMGB3	-0.3084425	0.084675912
TC2000007855.hg.1	SPO11	-0.309055	0.0512073
TC0100012544.hg.1	MMEL1	-0.30956	0.098341085
TC1300008289.hg.1	ZDHHC20	-0.310165	0.075486276
TC1700008938.hg.1	UBALD2	-0.31048	0.0512073
TC0400011018.hg.1	CXCL2	-0.31101	0.060475341
TC1600007235.hg.1	PLK1	-0.312165	0.035844342
TC0100016163.hg.1	ADAMTS4	-0.3123025	0.0512073
TC0900007848.hg.1	NXNL2	-0.31382	0.087398953
TC2000009970.hg.1	SIRPB1	-0.3141075	0.0512073
TC0100016406.hg.1	VAMP4	-0.3146525	0.051196291
TC1600009689.hg.1	PDZD9	-0.3147775	0.052350227
TC0200016099.hg.1	HJURP	-0.31479	0.0512073
TC0400011748.hg.1	CCNA2	-0.31505	0.071470684
TC2200007544.hg.1	BIK	-0.31601	0.067904095
TC0300008324.hg.1	PHLDB2; PLCXD2	-0.316175	0.093578588
TC0900012155.hg.1	MSANTD3-TMEFF1	-0.3162	0.06837185
TC0900006538.hg.1	JAK2	-0.3182325	0.059587735
TC1700008690.hg.1	PITPNC1	-0.31878	0.090975338
TC1400007443.hg.1	HSPA2	-0.319555	0.068605755
TC0X00010484.hg.1	COL4A6	-0.31956	0.03470232
TC2200006540.hg.1	USP18	-0.31957	0.094396878
TC0900008628.hg.1	CNTRL	-0.319635	0.057313125
TC1900011693.hg.1	ZNF429	-0.3210225	0.076769831
TC0100007102.hg.1	PADI1	-0.3213	0.093282551
TC1400009184.hg.1	TXNDC16	-0.321725	0.0512073
TC1900008561.hg.1	AKT1S1	-0.321745	0.0512073
TC0300008130.hg.1	COL8A1	-0.32194	0.081470333
TC0X00008747.hg.1	GABRQ	-0.322285	0.0512073

TC0700013224.hg.1	LMBR1	-0.3225425	0.094005923
TC0600007820.hg.1	CLPSL1	-0.32304	0.07498156
TC0300011172.hg.1	TNNC1	-0.3234675	0.099093194
TC0100018226.hg.1	RP5-1198O20.4; KLF17	-0.323965	0.099876826
TC0500013215.hg.1	LVRN	-0.32492	0.092203455
TC1400009329.hg.1	RTN1	-0.326135	0.095424527
TC1200012802.hg.1	PAN2; CNPY2	-0.3267475	0.087590101
TC0400012452.hg.1	FBXO8	-0.327055	0.059776557
TC0600009080.hg.1	CEP57L1	-0.3287375	0.092429672
TC0300012236.hg.1	MUC13	-0.3289875	0.072544283
TC1500006999.hg.1	NUSAP1	-0.33029	0.04611798
TC0300009795.hg.1	RTP1	-0.33171	0.095026515
TC2200007356.hg.1	KDELR3	-0.3317375	0.0512073
TC0300009136.hg.1	TM4SF1-AS1	-0.331775	0.096891836
TC0200015776.hg.1	RESP18	-0.33286	0.083456849
TC0X00008232.hg.1	ZCCHC12	-0.334035	0.085204682
TC1600011494.hg.1	ARL6IP1	-0.33503	0.025547592
TC0500009648.hg.1	N4BP3	-0.335065	0.072373494
TC0400009088.hg.1	GUCY1B3	-0.335105	0.033273608
TC1000008471.hg.1	KIF11	-0.337815	0.067091848
TC0100013369.hg.1	STMN1; MIR3917	-0.3397675	0.025111113
TC1200010050.hg.1	LMO3	-0.34021	0.031751474
TC1000009714.hg.1	PRKCQ	-0.341215	0.099626159
TC0900009561.hg.1	LINC01235	-0.3417175	0.043835359
TC0100007584.hg.1	PTPRU	-0.3433925	0.03483561
TC1500007699.hg.1	KIF23	-0.343505	0.033225252
TC0300008853.hg.1	TMEM108	-0.343995	0.092776123
TC0600011448.hg.1	LY6G6C	-0.346825	0.076298922
TC1700012285.hg.1	PRR11	-0.347455	0.045370227
TC0300012924.hg.1	VEPH1	-0.3487325	0.035844342
TC0300007221.hg.1	ZNF501	-0.3490375	0.095873626
TC0100011064.hg.1	CFH	-0.34994	0.075667099
TC0100016891.hg.1	KIF14	-0.3519375	0.067361676
TC1900011470.hg.1	UBE2S	-0.352095	0.0512073
TC1000012474.hg.1	NUTM2D	-0.35332	0.091429365
TC1900008384.hg.1	CALM3	-0.3537425	0.029506117
TC0200015204.hg.1	COL5A2	-0.3541775	0.028062459
TC0600014215.hg.1	GMDS	-0.35478	0.097654003
TC0800007665.hg.1	RP1	-0.3555125	0.043835359
TC1600007014.hg.1	NDE1; MIR484	-0.356095	0.021851241
TC1200007906.hg.1	XRCC6BP1	-0.35802	0.056350538
TC1600008986.hg.1	GNG13	-0.359775	0.062989335
TC0200012248.hg.1	FEZ2	-0.3600025	0.069665424
TC0900006522.hg.1	SLC1A1	-0.3603975	0.053192954
TC2000009996.hg.1	DUSP15	-0.3609825	0.064308903
TC1200007764.hg.1	ZNF385A	-0.3613875	0.037818217
TC0100007638.hg.1	SERINC2	-0.3620325	0.04974955
TC0800009094.hg.1	DENND3	-0.3629425	0.084587398
TC1000008482.hg.1	CEP55	-0.363885	0.087810386
TC0900009043.hg.1	GFI1B	-0.36441	0.073665224

TC0300006791.hg.1	KAT2B	-0.36519	0.085970286
TC0700007984.hg.1	VPS37D	-0.3655525	0.092203455
TC2200008036.hg.1	USP41	-0.366015	0.085583092
TC2100006784.hg.1	JAM2	-0.3662425	0.027089429
TC1500010422.hg.1	IDH2	-0.366955	0.027911185
TC0100007117.hg.1	ARHGEF10L	-0.3673125	0.0512073
TC1500010463.hg.1	PRC1	-0.36886	0.053535836
TC0900007064.hg.1	DNAJB5	-0.36981	0.034332503
TC2000006688.hg.1	SNAP25	-0.3702	0.043835359
TC1100010418.hg.1	KIF18A	-0.37055	0.033225252
TC0200009470.hg.1	HNMT	-0.3705625	0.025111113
TC0300012572.hg.1	CEP70	-0.37072	0.100493558
TC0100009307.hg.1	NTNG1	-0.372825	0.074271886
TC0800007389.hg.1	C8orf4	-0.3765325	0.067091848
TC0500012166.hg.1	CDC25C	-0.3765775	0.050357317
TC0100009324.hg.1	FAM102B	-0.3767975	0.035844342
TC2200006432.hg.1	BAGE5	-0.3771775	0.072373494
TC0200015951.hg.1	PID1	-0.3781625	0.030562683
TC1000008968.hg.1	ADRB1	-0.3794375	0.041665902
TC0X00009988.hg.1	ZMYM3	-0.3796	0.028797223
TC1900011103.hg.1	TULP2	-0.3796325	0.08475445
TC0X00010990.hg.1	SPANXC; SPANXD	-0.3819725	0.092203455
TC0X00006585.hg.1	SHROOM2	-0.3827325	0.0512073
TC0700013338.hg.1	GLCCI1	-0.3833825	0.069665424
TC0900009825.hg.1	DDX58	-0.3847625	0.079872125
TC1000008054.hg.1	PLAU	-0.3847675	0.01247524
TC0800012387.hg.1	DLC1	-0.3863775	0.062798851
TC0100017172.hg.1	IRF6	-0.3876825	0.095873626
TC0300009122.hg.1	CPA3	-0.3877775	0.098341085
TC1900009637.hg.1	CDKN2D	-0.388345	0.053535836
TC1200012143.hg.1	CIT; MIR1178	-0.3904475	0.043671166
TC0300012943.hg.1	LXN	-0.393295	0.076525154
TC1100010535.hg.1	PAMR1	-0.3955	0.028797223
TC2200008845.hg.1	SHISA8	-0.3964975	0.072628767
;Unmapped00000475.hg	PADI4	-0.3971975	0.046330913
TC1700012370.hg.1	TBC1D28	-0.3974025	0.0512073
TC0100014543.hg.1	DEPDC1	-0.398625	0.015650572
TC2000007341.hg.1	FAM83D	-0.4002525	0.015620196
TC0700010437.hg.1	RAPGEF5	-0.4010475	0.092203455
TC0700011066.hg.1	COBL	-0.4024625	0.066567516
TC1500007829.hg.1	LOXL1	-0.4047625	0.046873561
;Unmapped00000815.hg	RPS6KA1	-0.4104525	0.08475445
TC1900010802.hg.1	LIPE	-0.4109775	0.037818217
TC1600011362.hg.1	ACSM3	-0.41193	0.051196291
TC0500009097.hg.1	SYNPO	-0.412705	0.021865032
TC0800009465.hg.1	SPAG11B	-0.4131325	0.0512073
TC0100015509.hg.1	FAM72B	-0.4166	0.013312527
TC0100017094.hg.1	FAM72A	-0.416675	0.015497907
TC1100011285.hg.1	B4GAT1	-0.4178	0.019900199
TC1300008080.hg.1	TUBGCP3	-0.4182475	0.0512073

TC2000007015.hg.1	PYGB	-0.4185125	0.043671166
TC1200008650.hg.1	ASCL1	-0.4206325	0.056626913
TC0400007840.hg.1	CXCL1	-0.423435	0.019594536
TC0300011941.hg.1	MYH15	-0.4301125	0.065232968
TC0100009333.hg.1	GPSM2	-0.4321575	0.028797223
TC0300009531.hg.1	NLGN1	-0.43273	0.060248261
TC0200016417.hg.1	CENPA	-0.4337175	0.031580265
TC1200006520.hg.1	TSPAN9	-0.43516	0.029506117
TC1100010410.hg.1	BDNF	-0.4357825	0.0512073
TC0200012997.hg.1	TGFA	-0.4358225	0.0114496
TC1900009186.hg.1	ATP8B3	-0.4362475	0.019900199
TC1000007910.hg.1	COL13A1	-0.4364275	0.043835359
TC0200013008.hg.1	CD207	-0.436575	0.046873561
TC1400006540.hg.1	RNASE2	-0.436725	0.072373494
TC0900011947.hg.1	GLT6D1	-0.437285	0.094452601
TC0100017947.hg.1	OPN3	-0.44114	0.0114496
TC0600010057.hg.1	ACAT2	-0.441545	0.013312527
TC1400007566.hg.1	SMOC1	-0.442285	0.015797933
TC1600010532.hg.1	CDH8	-0.445015	0.039202312
TC1300009980.hg.1	LMO7	-0.4454125	0.043835359
TC1600010012.hg.1	PYDC1	-0.446225	0.033167433
TC1200008597.hg.1	GAS2L3	-0.44792	0.040005001
TC1700012380.hg.1	SPAG5	-0.449615	0.025547592
TC1100008069.hg.1	CNIH2	-0.450175	0.052143138
TC0200015432.hg.1	MPP4	-0.4515325	0.025547592
TC0900012163.hg.1	ORM2	-0.4516625	0.066567516
TC0100009723.hg.1	FAM72D	-0.4517125	0.015797933
TC0100007105.hg.1	PADI4	-0.4521975	0.01900165
TC1100013152.hg.1	SAA2; SAA2-SAA4; SAA4	-0.45341	0.043835359
TC0700012453.hg.1	SLC13A1	-0.45342	0.099876826
TC0600013190.hg.1	MOXD1	-0.4538425	0.0114496
TC0500009320.hg.1	HMMR	-0.4540375	0.011058322
TC0800012470.hg.1	TMEM71	-0.455225	0.031580265
TC2000009357.hg.1	PREX1	-0.4561825	0.0114496
TC0700012439.hg.1	AASS	-0.4564425	0.060248261
Unmapped00000049.hg	PADI3	-0.4591725	0.028923376
TC1100009433.hg.1	HYLS1	-0.460225	0.024899557
TC1500007392.hg.1	CCNB2	-0.470105	0.025954412
TC0100015550.hg.1	FAM72C	-0.4778625	0.009241112
TC1000010870.hg.1	SRGN	-0.4781925	0.059776557
TC0100015192.hg.1	PSRC1	-0.4793025	0.046498911
TC0100007103.hg.1	PADI3	-0.481445	0.034996871
TC1300006734.hg.1	MTUS2	-0.48247	0.0512073
TC0100014528.hg.1	DIRAS3	-0.4829575	0.043835359
TC1400009248.hg.1	DLGAP5	-0.4836775	0.008102
TC0200014672.hg.1	NR4A2	-0.4869225	0.072791484
TC1700012458.hg.1	CEP112	-0.490425	0.094919693
TC0X00007325.hg.1	RIBC1	-0.4919725	0.023149911
TC1200012853.hg.1	NOS1	-0.496215	0.099093194
TC1200012753.hg.1	KLRC2	-0.496325	0.060691477

TC0800009428.hg.1	XKR5	-0.497885	0.035016894
TC0100013028.hg.1	EPHA2	-0.5014625	0.011058322
TC0200009539.hg.1	ARHGAP15	-0.5020075	0.087810386
TC0100014995.hg.1	CNN3	-0.5027075	0.011058322
TC0500007665.hg.1	CCNB1	-0.5057425	0.011058322
TC0600007266.hg.1	HIST1H1C	-0.514315	0.038822528
TC0100016831.hg.1	ASPM	-0.51919	0.018161365
TC0200013913.hg.1	IL1A	-0.5222225	0.009877926
TC1500009200.hg.1	TTBK2	-0.522465	0.0512073
TC0700012276.hg.1	DOCK4	-0.522535	0.031930039
TC1400007201.hg.1	CDKN3	-0.5248175	0.018847454
TC0200015266.hg.1	TMEFF2	-0.5277475	0.024710658
TC1500008983.hg.1	SLC12A6	-0.5281	0.027911185
TC0500013309.hg.1	ESM1	-0.5346275	0.054474791
TC1500007739.hg.1	THSD4	-0.5365425	0.070749101
TC1000009155.hg.1	DMBT1	-0.5376925	0.008102
TC0800012308.hg.1	ADHFE1; C8orf46	-0.539645	0.062852995
TC0400006951.hg.1	CD38	-0.55091	0.100493558
TC1400009175.hg.1	NID2	-0.5514475	0.009241112
TC2000007083.hg.1	ID1	-0.5577525	0.015620196
TC0100014585.hg.1	NEGR1	-0.5586825	0.018616939
TC0200009739.hg.1	GALNT5	-0.5653375	0.017759987
TC1700009730.hg.1	RCVRN	-0.570695	0.083270988
TC0100016694.hg.1	IVNS1ABP	-0.57712	0.008102
TC1500010184.hg.1	BCL2A1	-0.5806275	0.01257972
TC0200013298.hg.1	ST3GAL5	-0.5837325	0.041776133
TC0600007694.hg.1	B3GALT4	-0.5951175	0.005637494
TC0500010635.hg.1	HMGCS1	-0.60603	0.008102
TC1200012856.hg.1	VSIG10	-0.6209125	0.032071873
TC0500008777.hg.1	KIF20A	-0.6344775	0.005200956
TC0100017216.hg.1	NEK2	-0.6460625	0.009144768
TC1800007340.hg.1	DCC	-0.6596625	0.034996871
TC1000006782.hg.1	ECHDC3	-0.694735	0.033167433
TC0800009782.hg.1	CSGALNACT1	-0.69647	0.009241112
TC1900011467.hg.1	IL11	-0.7352325	0.008102
TC0800012006.hg.1	PTK2	-0.75084	0.019900199
TC1200007626.hg.1	METTL7A	-0.792345	0.0114496
TC0200015350.hg.1	RFTN2	-1.16008	0.009877926
